# Dopamine D1-like and Angiotensin II Type 1 Receptors Counter-Regulate Autophagy and Cell Proliferation in Rat Embryonic Thoracic Vascular Smooth Muscle Cells

**DOI:** 10.3390/ijms27156784

**Published:** 2026-07-29

**Authors:** Hewang Lee, Amy Lu, Waleed N. Qaddumi, Bibhas Amatya, Jacob Polzin, Maithri Verma, Raisha C. Cadme, Robin A. Felder, Ines Armando, Jeffrey B. Kopp, Pedro A. Jose

**Affiliations:** 1 Department of Medicine, Division of Kidney Disease & Hypertension, The George Washington University School of Medicine & Health Sciences, Washington, DC 20052, USA; lih@gwu.edu (H.L.); amylu2027@u.northwestern.edu (A.L.); waleed.qaddumi@gwmail.gwu.edu (W.N.Q.); bibhasamatya78@gwmail.gwu.edu (B.A.); polzin@gwmail.gwu.edu (J.P.); maithri@ucsb.edu (M.V.); raishalorena@gmail.com (R.C.C.); iarmando@gwu.edu (I.A.); 2 Department of Pharmacology and Physiology, The George Washington University School of Medicine & Health Sciences, Washington, DC 20052, USA; 3 Department of Pathology, University of Virginia Health Sciences Center, Charlottesville, VA 22908, USA; raf7k@virginia.edu; 4 Department of Pediatrics, Georgetown University Medical Center, Washington, DC 20007, USA; 5 Center for Molecular Physiology Research, Children’s National Research Institute, Washington, DC 20010, USA; 6 Independent Researcher, 2324 North Apple Lane, Pocatello, ID 83204, USA; koppjb@gmail.com

**Keywords:** autophagy, D1-like receptors, Ang II type 1 receptor, cyclic AMP, mTOR, proliferation, VSMC

## Abstract

Vascular smooth muscle cells (VSMCs), the contractile cells in the tunica media of blood vessels, maintain vascular tone. The proliferation of VSMCs is an important feature of vascular remodeling that contributes to the regulation of blood pressure. Autophagy, an intracellular self-degrading process that delivers cytoplasmic constituents to lysosomes, plays a vital role in VSMC proliferation. This is regulated by the dopaminergic and renin–angiotensin systems but their interplay in their regulation of autophagy in VSMCs is not well-understood. In rat VSMCs, fenoldopam (Fen), a dopamine D1-like receptor agonist, increased autophagy, as determined by the increase in the protein expressions of microtubule-associated protein 1 light chain (LC)3-II and beclin-1 (BECN1), in a time- and concentration-dependent manner. Conversely, angiotensin II (Ang II), the endogenous Ang II type 1 receptor (AT_1_R) agonist, decreased the protein expression of LC3-II and BECN1, also in a time- and concentration-dependent manner. The production of cyclic adenosine monophosphate (cAMP) and autophagic LC3-II puncta in VSMCs were increased by Fen and decreased by Ang II. Pre-treatment of VSMCs with Rp-cAMPS, a protein kinase A inhibitor, prevented the Fen-mediated increase and the Ang II-mediated decrease in LC3-II protein expression. Fen decreased, whereas Ang II increased the phosphorylation of P70S6K, a direct downstream mammalian target of rapamycin (mTOR). The inhibitory effect of Fen and stimulatory effect of Ang II on P70S6K phosphorylation were prevented by Rp-cAMPS. Ang II also decreased the Fen-mediated increase in cAMP production, while Fen attenuated the Ang II-mediated increase in cell proliferation, a response that occurs downstream of autophagy. Moreover, Ang II prevented the Fen-mediated inhibition of cell proliferation, an effect that was blocked by losartan, an AT_1_R antagonist. These results demonstrate that Fen and Ang II counter-regulate autophagy and proliferation of VSMCs via the mTOR pathway, which is cAMP-dependent.

## 1. Introduction

The cellular proliferation of vascular smooth muscle cells (VSMCs) is pivotal in supporting their primary physiological functions, including the maintenance of vascular resistance and capacitance, and, ultimately, vascular tone [1]. VSMC proliferation also contributes to the pathogenesis of vascular diseases, such as hypertension and atherosclerosis [1,2]. Of particular relevance is that the dopaminergic and renin–angiotensin systems are among the major regulators of VSMC function and dysfunction (*vide infra*).

Dopamine, a well-known neurotransmitter in the central nervous system (CNS), also exerts widespread effects in non-neuronal cells, including arterial VSMCs [3,4,5,6]. Dopamine receptors are grouped into two subfamilies: the D1-like receptors (D_1_R and D_5_R) and D2-like receptors (D_2_R, D_3_R, and D_4_R). The D1-like receptors couple to the stimulatory G protein, Gαs, to stimulate adenylyl cyclase activity, whereas the D2-like receptors couple to the inhibitory G protein, Gαi, to inhibit adenylyl cyclase activity [3,4,5,6,7]. D1-like receptors, expressed in conduit and resistance vessels [3,4,5], are important in vasorelaxation, suppression of VSMC proliferation, and regulation of blood pressure [7,8].

The autocrine/paracrine renin–angiotensin–aldosterone system can increase VSMC proliferation, vascular tone, and blood pressure [9,10]. Angiotensin II (Ang II), the most potent vasoactive component in this system, exerts its action through its receptors, mainly Ang II type I receptor (AT_1_R) and AT_2_R [9,10]. AT_1_R is mainly linked to Gαq/11 and to Gαi as well in VSMCs [9,10], whereas AT_2_R activates nitric oxide/cGMP through Gαi/_O_ in VSMCs [9]. However, in VSMCs, the principal effect of Ang II is the induction of vascular contraction, increasing vascular tone and blood pressure through the activation of AT_1_R [9].

Autophagy is an evolutionarily conserved degradative process that preserves intracellular homeostasis by selectively encapsulating damaged macromolecules and dysfunctional organelles within double-membrane vesicles for lysosomal degradation and by non-selectively degrading cellular components to fuel intracellular stabilization [11,12]. The autophagic process substantially participates in the regulation of various VSMC functions, including VSMC proliferation [2]. However, within the context of the dopaminergic and angiotensin systems, the connection between these neurohormonal systems and the autophagy-mediated pathway that regulates VSMC proliferation still remains unclear.

D1-like receptors upregulate autophagy in renal proximal tubule cells, kidney cortices [13], and mouse bone marrow-derived macrophages [14]. Yet, how D1-like receptors contribute to the underlying mechanisms of autophagy in the vasculature is poorly understood. In VSMCs, the activation of AT_1_R has been reported to have a bidirectional effect on autophagy, where it can either decrease [15] or increase [16] its activity. Additionally, in renal proximal tubule cells, Ang II has the ability to increase [17] or have no effect [18] on autophagy. These highlight the variable effects of Ang II on autophagic processes that could be attributed to the compartmentalization of signaling cascades across different cellular microenvironments [19]. In several organs, including the kidney, D1-like receptors and AT_1_Rs antagonize each other’s effects such as those related to inflammation [4], sodium excretion [20], and blood pressure regulation [3,21,22]. For example, the natriuretic effect of D1-like receptors is enhanced when Ang II production is reduced or when AT_1_R is blocked [3]. Despite their individual actions, the crosstalk between D1-like receptors and AT_1_Rs in VSMCs, as related to autophagy, has not been fully characterized.

D1-like receptors are natively coupled to Gαs, which stimulates adenylyl cyclase and elevates cAMP levels [3,4,5,6,7,8]; AT_1_R exhibits diverse G-protein coupling profiles, including Gαi, through which AT_1_R effectively inhibits adenylyl cyclase activity, leading to a suppression of Gαs-induced cAMP production [9,10]. Thus, to understand the fine-tuning of intracellular signaling and autophagic homeostasis in the vasculature, in this study, we aimed to investigate the multifaceted effects and interactions of D1-like receptors and AT_1_Rs that shape the autophagic dynamics and cell proliferative responses in VSMCs.

## 2. Results

### 2.1. D1-like Receptors Increase Autophagy in VSMCs

**Figure 1:**
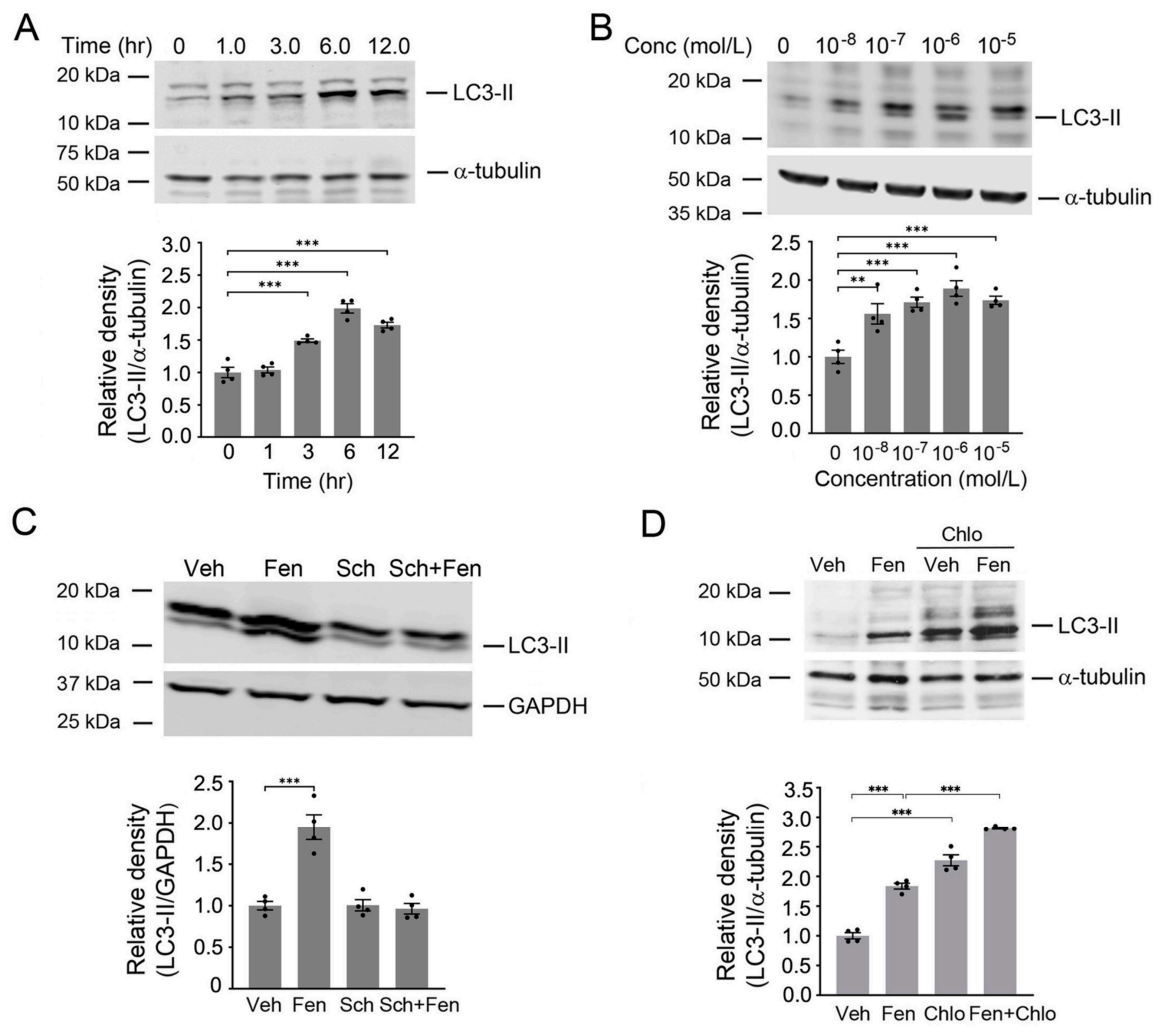
**D1-like receptor-mediated increase in autophagy in VSMCs**. (**A**) VSMCs were treated with Fen (1 µmol/L) at the indicated times. (**B**) VSMCs were treated with Fen for 6 h, at the indicated concentrations. (**C**) Effect of Sch22390 (Sch, 1.0 µmol/L), a D1-like receptor antagonist, on the Fen-mediated increase in LC3-II protein expression in VSMCs. VSMCs were treated as indicated for 6 h. (**D**) Effect of chloroquine (Chlo, 10 µmol/L), an autophagy inhibitor, on the Fen-mediated increase in LC3-II protein expression in VSMCs. The immunoblots (**A**–**D**) were quantified and expressed as the density ratio of LC3-II over loading control (α-tubulin or GAPDH), then normalized by the control (vehicle). Veh, vehicle; Fen, fenoldopam; Sch, Sch23390; Chlo, chloroquine; hr, hour. *n* = 4/Group, ** *p* < 0.01; *** *p* < 0.001. One-way ANOVA, Bonferroni multiple comparison test (**A**–**D**).

In VSMCs, the D1-like receptor agonist, fenoldopam (Fen) [3,5,7,8,13,21,23], increased the protein expressions of LC3-II (Figure 1A,B) and BECN1 (Supplementary Figure S1A,B) in a time- and concentration-dependent manner, with the optimal time of 6 h and concentration of 1 μmol/L.

To determine whether the effect of Fen was through D1-like receptors, the VSMCs were pre-treated with the D1-like receptor antagonist, Sch23390 [5,7,8,13,21,23] (1 μmol/L, 30 min), before the addition of Fen, Sch23390 (Figure 1C and Supplementary Figure S2A) or the combined silencing of *Drd1* and *Drd5* with their specific siRNAs (Supplementary Figure S2B). The latter treatment prevented the ability of Fen (agonist for both D_1_R and D_5_R) (1 μmol/L, 6 h) to increase LC3-II protein expression. By contrast, the silencing of either *Drd1* (Supplementary Figure S3A) or *Drd5* (Supplementary Figure S3B), by their specific siRNAs, attenuated and did not completely prevent the stimulatory effect of Fen (1 μmol/L, 6 h) on LC3-II protein expression, suggesting that the effect of Fen was through the activation of both D1-like receptors.

Consistent with these results, LC3-II puncta were increased by Fen (1 μmol/L, 6 h) (Supplementary Figure S4). The Fen-mediated increase in LC3-II puncta was prevented when the cells were pretreated with the D1-like receptor antagonist, Sch (1 μmol/L). However, the administration of Sch alone did not have any effect on LC3-II puncta (Supplementary Figure S4).

To determine whether the Fen-mediated increase in LC3-II protein expression was due to an increase in autophagic flux or a decrease in the degradation process, the VSMCs were treated with chloroquine (10 μmol/L), an inhibitor of autophagosome–lysosome fusion [13,24] in the presence or absence of Fen (1 μmol/L). We found that LC3-II protein expression was increased by either Fen or chloroquine, which was further increased by the combination of Fen and chloroquine (Figure 1D). These results indicate that the D1-like receptor-mediated increase in LC3-II protein expression is due to the increase in autophagosome–lysosome fusion (i.e., autophagic flux) rather than the blockade of protein degradation [13].

### 2.2. AT_1_R Decreases Autophagy in VSMCs

**Figure 2:**
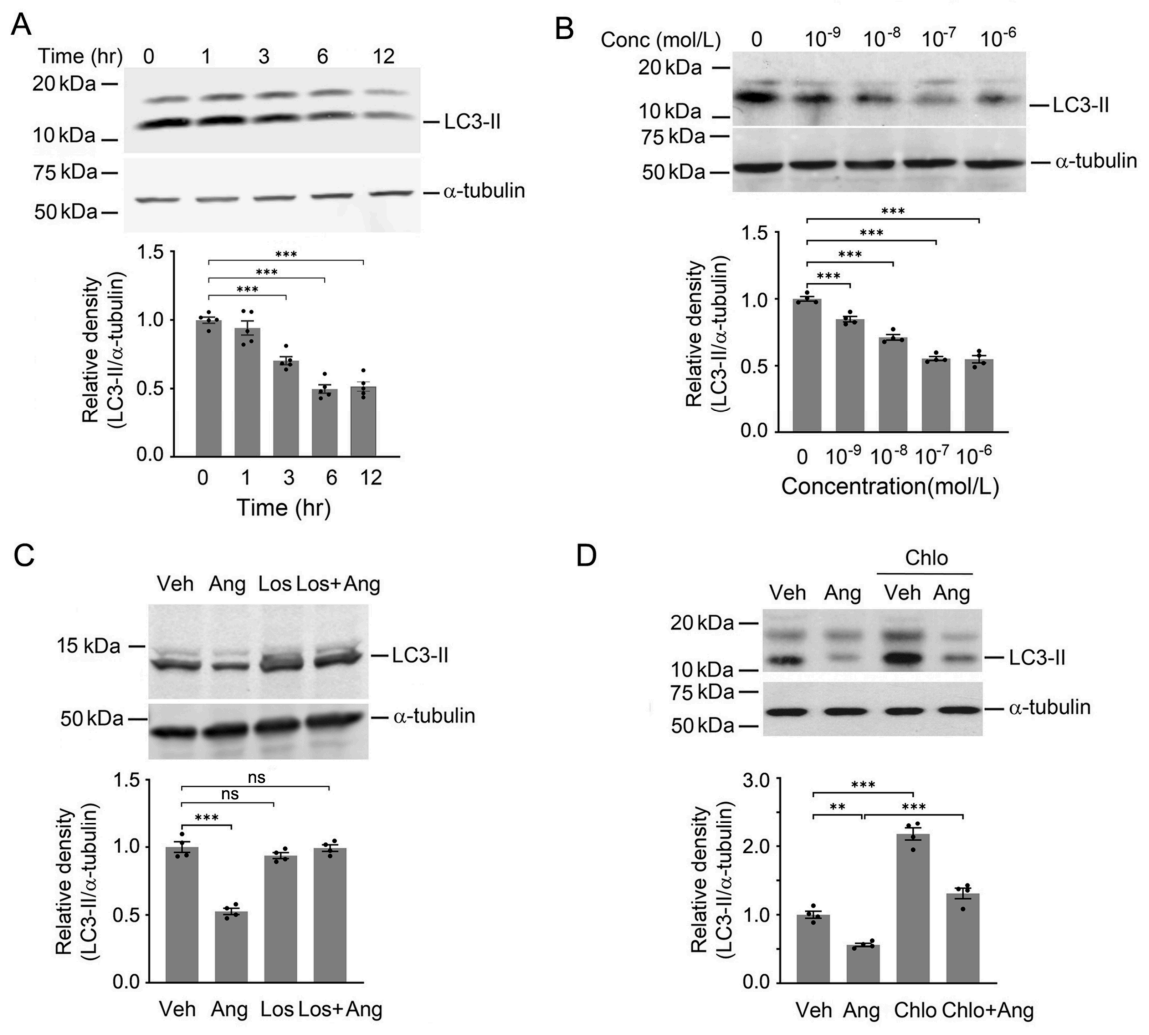
**AT_1_R-mediated decrease in autophagy in VSMCs**. (**A**) VSMCs were treated with Ang II (100 nmol/L) at the indicated times. (**B**) VSMCs were treated with Ang II for 6 h, at the indicated concentrations. (**C**) Effect of losartan (Los, 10 µmol/L), an AT_1_R antagonist, on the Ang II-mediated decrease in LC3-II protein expression in VSMCs. (**D**) Effect of chloroquine (Chlo, 10 µmol/L), an autophagy inhibitor, on the Ang II-mediated decrease in LC3-II protein expression in VSMCs. The immunoblots (**A**–**D**) were quantified and expressed as the density ratio of LC3-II over α-tubulin, then normalized by vehicle or control (0 h or 0 mol/L). Veh, vehicle; Ang, Ang II; Los, losartan; Chlo, chloroquine; hr, hour. *n* = 4–5/Group, ** *p* < 0.01, *** *p* < 0.001, ns, not significant. One-way ANOVA, Bonferroni multiple comparison test (**A**–**D**).

In VSMCs, Ang II decreased the protein expression of LC3-II (Figure 2A,B) and BECN1 (Supplementary Figure S5A,B) in a time- and concentration-dependent manner, with the maximal effect at 6 h and concentration of 100 nmol/L. The Ang II-mediated reduction in autophagy in VSMCs is consistent with a recent study showing that Ang II decreases the number of autophagic vacuoles in skeletal muscles [25].

To determine whether the Ang II effect was through the AT_1_R, the VSMCs were pre-treated with losartan, a specific AT_1_R antagonist [21] (10 μmol/L, 30 min before the addition of Ang II). Losartan, by itself, had no effect, but prevented the inhibitory effect of Ang II on LC3-II protein expression (Figure 2C) and the formation of LC3-II cytoplasmic puncta (Supplementary Figure S6). Consistent with the effect of the pharmacological inhibition of AT_1_R by losartan, the silencing of AT_1a_R by specific *Agtr1a* siRNA also prevented the Ang II-mediated inhibition of LC3-II protein expression (Supplementary Figure S7), proving that the Ang II’s inhibitory effect on LC3-II expression was via the stimulation of AT_1_R.

To determine further whether the AT_1_R-mediated decrease in LC3-II protein expression was due to a decrease in autophagic flux or an increase in lysosomal degradation, the VSMCs were treated with chloroquine [13,24] in the presence or absence of Ang II (100 nmol/L). We found that the autophagy inhibitor chloroquine, by itself, increased the expression of LC3-II protein. In the presence of chloroquine, the degree of the Ang II-mediated decrease in LC3-II expression was less than Ang II alone (Figure 2D), indicating that the Ang II-mediated decrease in LC3-II protein expression is mainly caused by a decrease in autophagic flux.

### 2.3. D1-like Receptors Increase Autophagy Through cAMP/PKA Pathway in VSMCs

**Figure 3:**
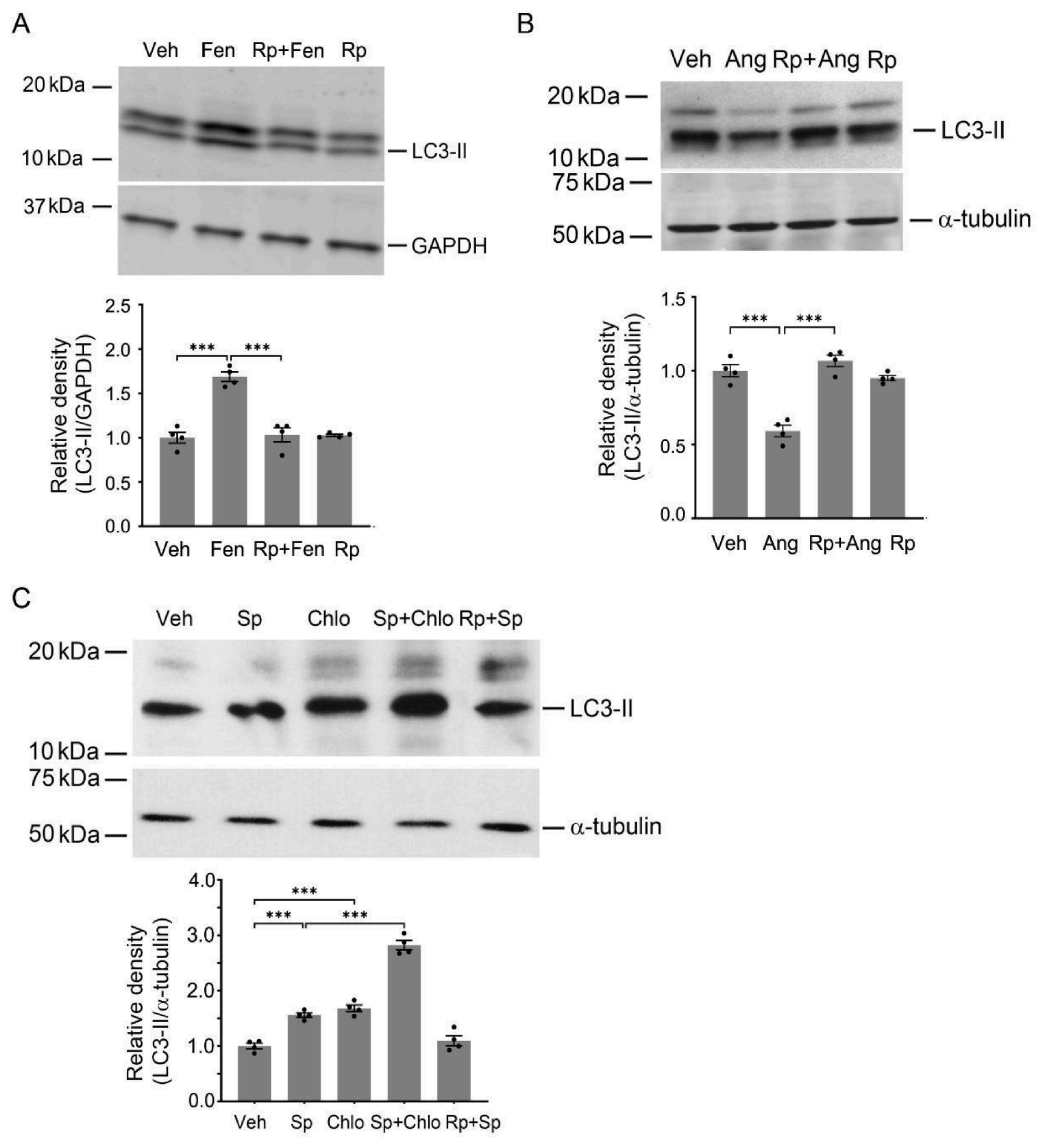
**D1-like receptor-mediated increase in autophagy is dependent on cAMP/PKA signaling in VSMCs**. (**A**) VSMCs were treated with vehicle (Veh), Fen (10^−6^ M), Rp-cAMPS (Rp, 50 µmol/L), or their combination (Fen + Rp) for 6 h. *n* = 4/Group, *** *p* < 0.001. One-way ANOVA, Bonferroni multiple comparison test. (**B**) The VSMCs were treated with vehicle (Veh), Ang II (10^−7^ M) (Ang), Rp-cAMPS (Rp, 50 µmol/L), or their combination (Ang + Rp) for 6 h. n = 4/Group, *** *p* < 0.001, one-way ANOVA, Bonferroni multiple comparison test. (**C**) The VSMCs were treated with Veh or Sp-cAMPS (Sp, 1.0 µmol/L) for 6 h. In the final 2 h of incubation, Veh or chloroquine (Chlo, 10 µmol/L) was added into the incubation medium. *n* = 4/Group, *** *p* < 0.001. One-way ANOVA, Bonferroni multiple comparison test. Veh, vehicle; Fen, fenoldopam; Rp, Rp-cAMPs; Sp, Sp-cAMPS; PBS, phosphate-buffered saline; Chlo, chloroquine.

D1-like receptors, which are coupled to Gαs, stimulate cAMP production [5,6,22], and activate protein kinase A (PKA) in the kidney [5,13,26]. Several studies have demonstrated that PKA is capable of regulating multiple steps in the autophagic process [19,27]. As expected, Fen increased cAMP production in VSMCs that was prevented by the D1-like receptor antagonist, Sch23390, which by itself had no effect (Supplementary Figure S8A). We therefore determined whether the Fen-mediated increase in autophagy was cAMP/PKA-dependent. We found that the Fen-mediated increase in LC3-II protein expression was prevented by Rp-cAMPS, a PKA inhibitor [13] (Figure 3A). When used alone, Rp-cAMPS had no effect on LC3-II protein expression (Figure 3A), proving that the D1-like receptor-mediated increase in autophagy is cAMP/PKA pathway-dependent.

### 2.4. AT_1_R Decreases Autophagy Through the cAMP/PKA Pathway in VSMCs

AT_1_R classically couples to Gαq/_11_, activates phospholipase C, and increases cytosolic Ca^2+^ and inositol 1,4,5-trisphosphate (IP3) levels [5,20,28], which has the ability to decrease autophagy [15]. AT_1_R also directly activates Gαi and inhibits adenylyl cyclase activity, which decreases cAMP production [10,29]. However, Ang II, by stimulation of prostaglandin synthesis, induces a transient (1–2 min) increase in cAMP production in VSMCs [30]. The AT_1_R-mediated stimulation of PKA signaling has been observed in Ang II-induced proteasome activation in T-helper cells [31]. However, in our study, using VSMCs, Ang II inhibited the forskolin-induced cAMP production that was reversed by losartan, which by itself had no effect (Supplementary Figure S8B). These results show that the activation of AT_1_R impairs cAMP/PKA signaling in VSMCs.

We next investigated whether the Ang II-mediated decrease in autophagy in VSMCs is dependent on the inhibition of the cAMP/PKA pathway. We found that Ang II decreased LC3-II protein expression and the Ang II-mediated decrease in LC3-II expression was prevented by pre-treatment with Rp-cAMPS, a PKA inhibitor (Figure 3B), similar to that caused by losartan (Figure 2C), and silencing of AT_1_aR by specific *Agtr1a* siRNA (Supplementary Figure S7). However, treatment with Rp-cAMPS, by itself, did not alter the level of LC3-II protein expression (Figure 3B). Treatment with Sp-cAMPS, a PKA activator [13,32], increased LC3-II protein expression that was prevented by pretreating the cells with the PKA inhibitor, Rp-cAMPS (Figure 3C) [13]. Chloroquine, an autophagy inhibitor, increased further the Sp-cAMPS-mediated increase in LC3-II protein expression (Figure 3C), indicating PKA is capable of inducing autophagy.

To dissect the role of PKA in the Ang II-mediated LC3-II protein expression, we performed PKA activity assay (Supplementary Figure S9). As expected, Rp-cAMPS, a competitive antagonist that prevents endogenous cAMP from activating PKA, indeed decreased PKA activity. The combination of Rp-cAMPS and Ang II further decreased PKA activity (Supplementary Figure S9), which seems paradoxical to the combination effect on autophagy that Rp-cAMPS prevented the Ang II-mediated decrease in LC3-II protein expression (Figure 3B). This could be taken to indicate that the Ang II-mediated decrease in autophagy was not the result of a simple linear reduction in cAMP-PKA signaling but rather indicative of a branched signaling network.

### 2.5. AT_1_R and D1-like Receptors Counter-Regulate cAMP Production and Autophagy in VSMCs

Since AT_1_R-mediated LC3-II protein expression was not simply a linear relationship from Gαi-cAMP-PKA to autophagy (Figure 3B and Supplementary Figure S9), and D1-like receptors and AT_1_R have an opposing effect in the renal regulation of blood pressure [5,20,21,22], we next investigated whether these two systems counter-regulate each other to fine-tune autophagy in VSMCs.

**Figure 4:**
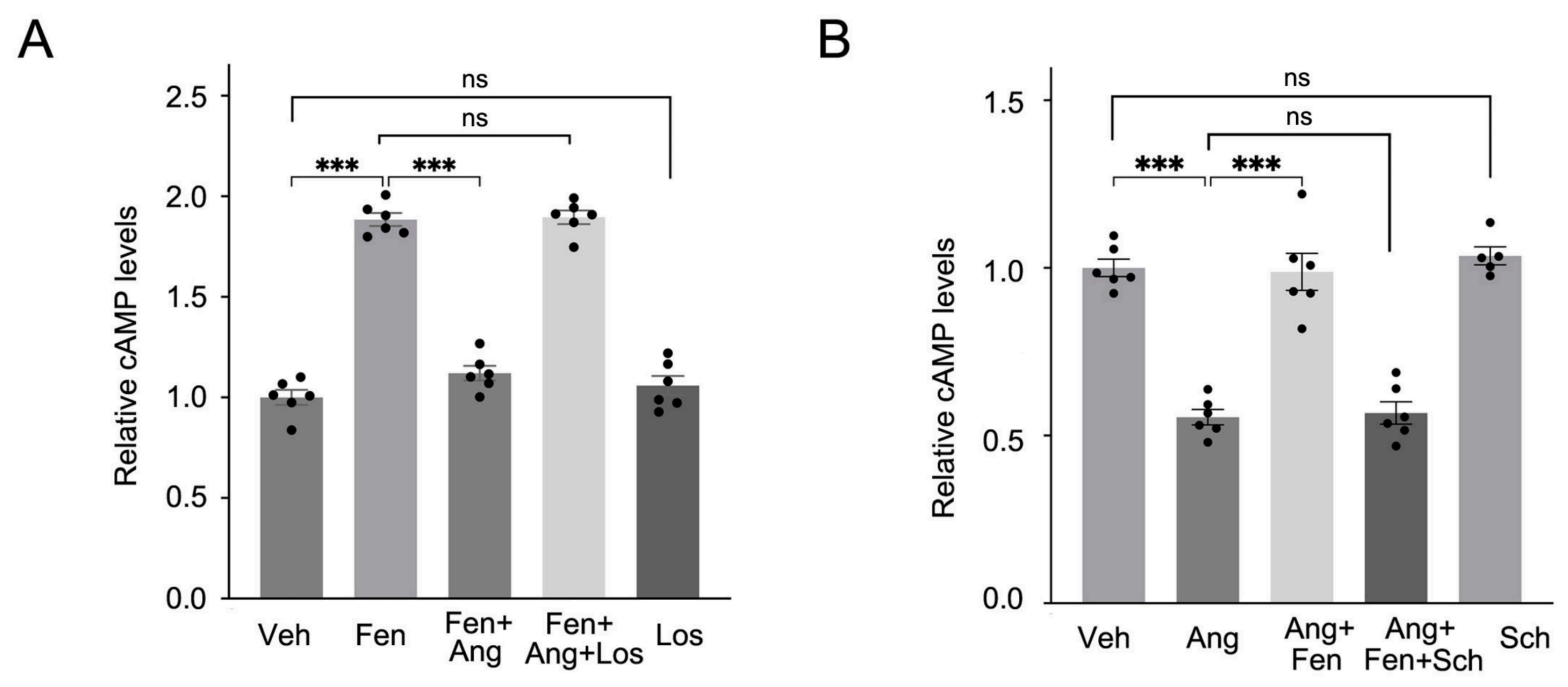
**Counter-regulatory effect of AT_1_R and D1-like receptors on cAMP production in VSMCs**. (**A**) Pre-treatment of VSMCs with Ang II (Ang, 100 nmol/L, 15 min) prevented the Fen (1 μmol/L, 15 min)-mediated increase in cAMP production (Fen + Ang), that was restored by the addition of Los (10 µmol/L), an AT_1_R antagonist. (**B**) Pre-treatment of VSMCs with Fen (1 μmol/L, 15 min) prevented the Ang II (100 nmol/L, 15 min)-mediated decrease in cAMP production (Ang + Fen) in VSMCs, that was restored by the addition of Sch (1 µmol/L), D_1_-like receptor antagonist. (**A**,**B**) Fen, fenoldopam; Sch, Sch23390; Ang, Ang II; Los, losartan; Veh, vehicle. *n* = 6/Group, *** *p* < 0.001, ns, not significant. One-way ANOVA, Bonferroni multiple comparison test.

**Figure 5:**
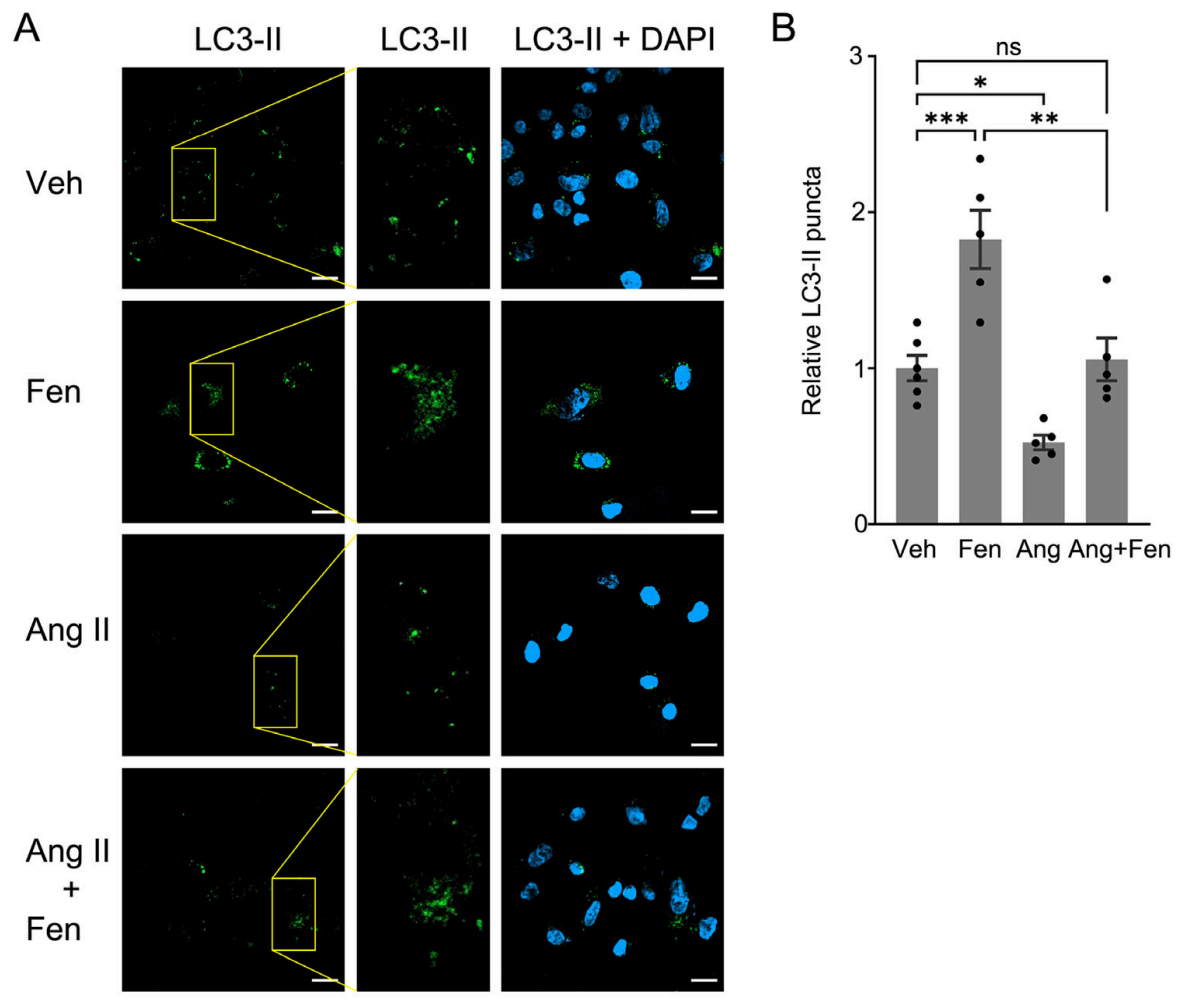
**Fenoldopam (Fen) increases whereas Ang II decreases LC3-II puncta in VSMCs**. (**A**) VSMCs were treated with Veh (vehicle), Fen (fenoldopam, 1.0 µmol/L, 12 h), Ang II (100 nmol/L, 12 h) or Fen + Ang II (combination of Fen and Ang II, 12 h), as indicated. Representative immunofluorescence images show endogenous LC3-II (green). Blue, nuclei (DAPI). Ang II, angiotensin II. Scale bars, 10 µm. (**B**) The number of LC3-II puncta was counted by ImageJ (Version 1.54d). The number of cells were counted manually by number of nuclei (cells on the edges were excluded for analysis). The number of LC3-II puncta were divided by the number of cells, and each group was normalized by the average number of the Veh group. Ang, angiotensin II. *n* = 5–6/Group, * *p* < 0.05, ** *p* < 0.01, *** *p* < 0.001, ns, not significant. One-way ANOVA, Bonferroni multiple comparison test.

**Figure 6:**
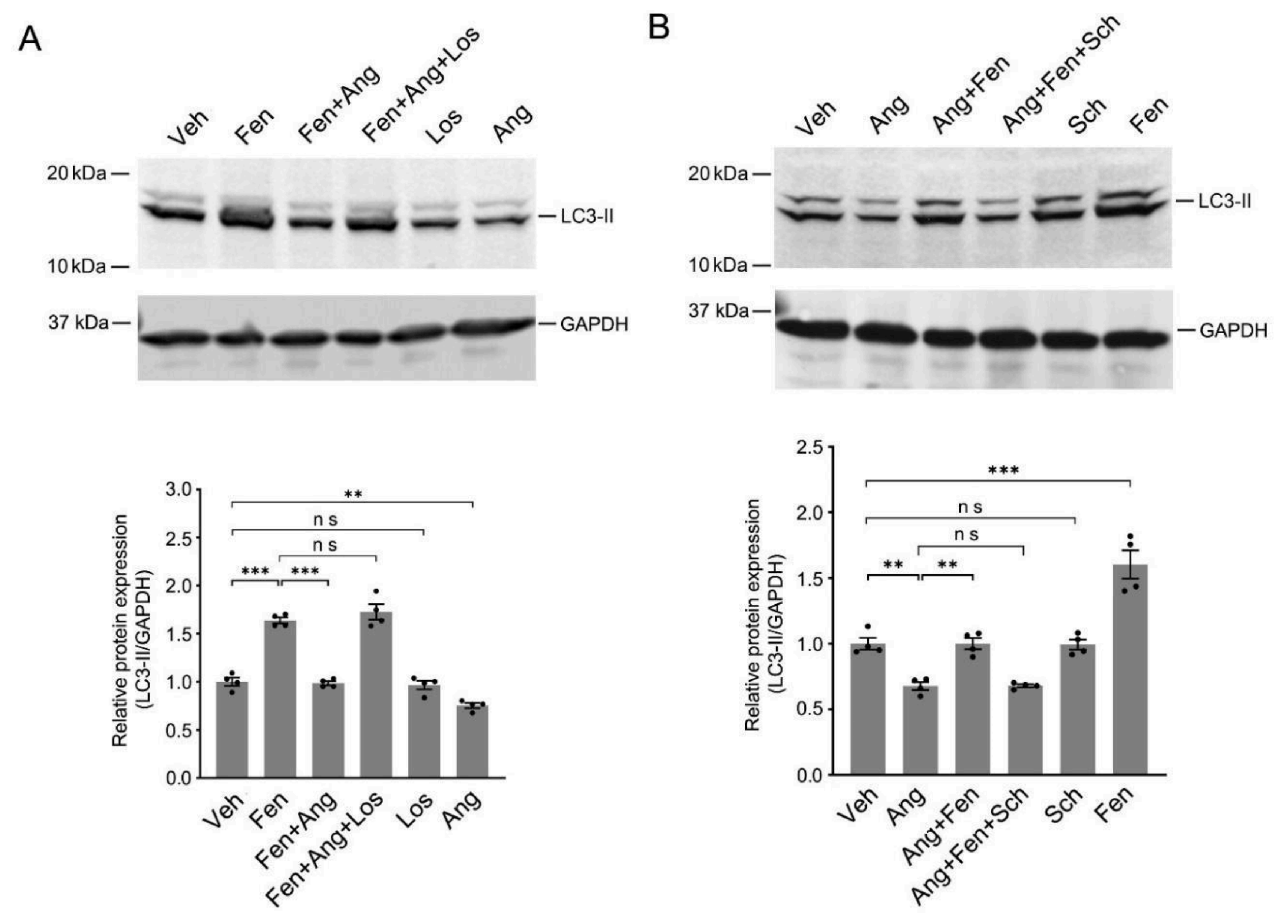
**Counter-regulatory effect of AT_1_R and D1-like receptors on LC3-II protein expression in VSMCs**. (**A**) VSMCs were treated with Fen (1 μmol/L), Ang II (Ang) (100 nmol/L), losartan (Los, 10 μmol/L) or their combination, as indicated for 6 h. The ability of Ang II to prevent the Fen-mediated increase in LC3-II protein expression was abolished by pre-treatment with the AT_1_R antagonist, Losartan (Los). Los alone had no effect but Ang, by itself, decreased LC3-II expression. (**B**) The VSMCs were treated with Ang (100 nmol/L), Fen (1 μmol/L), Sch23390 (Sch,1 μmol/L) or their combination, as indicated for 6 h. The ability of Fen to prevent the Ang-mediated decrease in LC3-II protein expression was abolished by pre-treatment with the D_1_-like receptor antagonist, Sch. Sch alone had no effect but Fen, by itself, increased LC3-II protein expression, as in Figure 1A–D and Figure 4A. (**A**,**B**) Veh, vehicle; Fen, fenoldopam; Ang, angiotensin II; Sch, Sch23390. *n* = 4/Group, ** *p* < 0.01, *** *p* < 0.001, ns, not significant. One-way ANOVA, Bonferroni multiple comparison test.

Indeed, Ang II prevented the Fen-mediated increase in cAMP concentration that was abolished by pre-treatment with losartan, an AT_1_R antagonist (Figure 4A), which by itself, had no effect (Figure 4B). Conversely, Fen counteracted the Ang II-mediated decrease in cAMP production, an effect that was abolished by pre-treatment with Sch23390, a D1-like receptor antagonist, which by itself had no effect (Figure 4B). Consistent with these opposing effects of Fen and Ang II on cAMP production, Ang II prevented the Fen-mediated increase in cytoplasmic LC3-II puncta formation, visualized by confocal microscopy (Figure 5), and an increase in LC3-II protein expression, quantified by immunoblotting (Figure 6).

The Ang II-mediated inhibition of the Fen-mediated increase in LC3-II protein expression was abolished when the VSMCs were pretreated with losartan (AT_1_R antagonist), which, by itself, had no effect (Figure 6A). By contrast, the Fen-mediated prevention of the decrease in LC3-II caused by Ang II was abolished when cells were pretreated with Sch23390 (D1-like receptor antagonist), which, by itself, had no effect (Figure 6B).

### 2.6. Mammalian Target of Rapamycin (mTOR) Dependence of D1-like Receptor- and AT_1_R-Mediated Regulation of Autophagy in VSMCs

**Figure 7:**
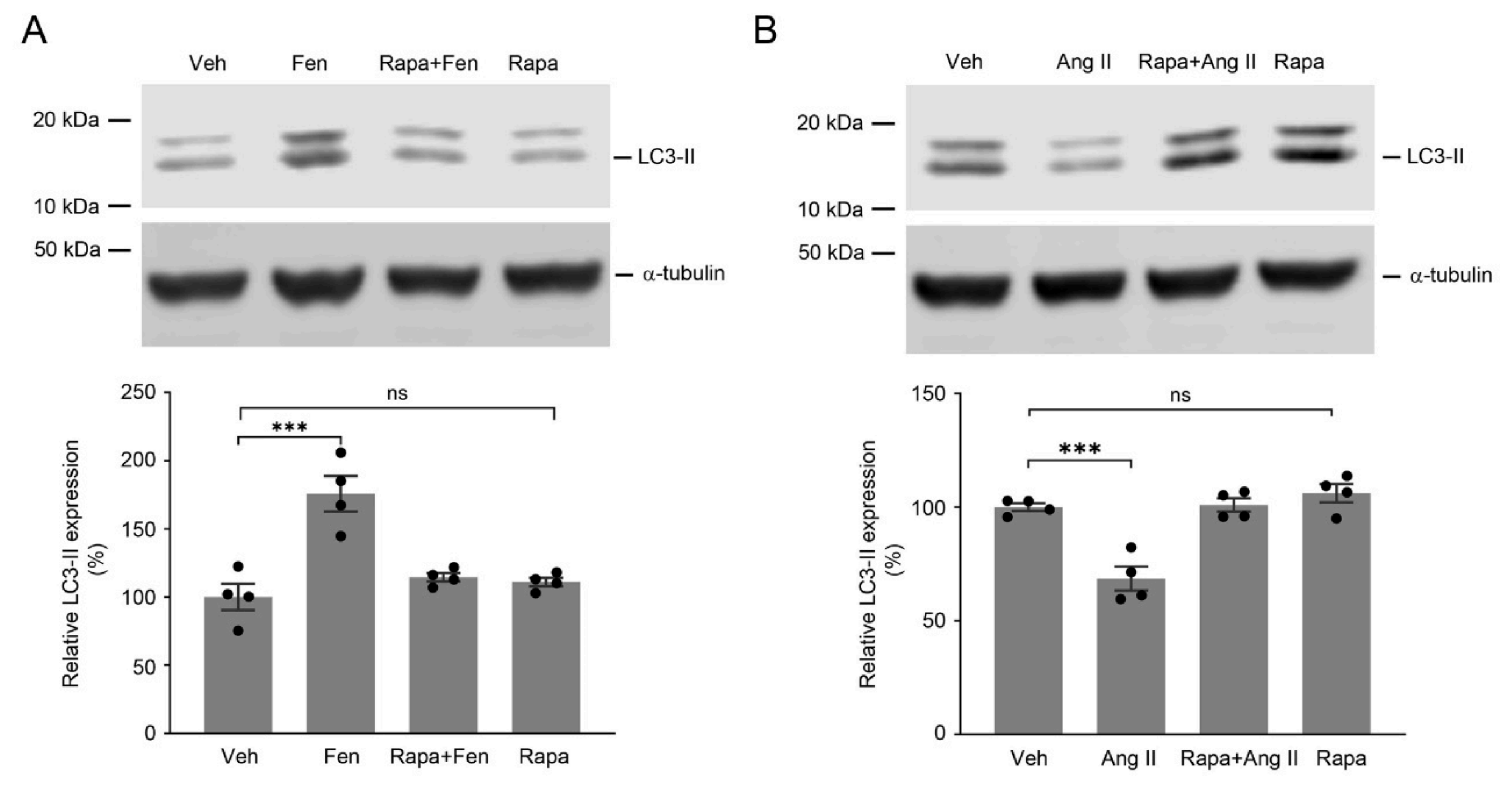
**Inhibition of mTOR by rapamycin prevented Fen-** (**A**) **and Ang II-** (**B**) **mediated regulation of autophagy in VSMCs**. VSMCs were treated with vehicle (Veh), Fen (1 μmol/L) or Ang II (Ang, 100 nmol/L), or their combination with rapamycin (Rapa, 100 nmol/L), as indicated, for 6 h. Rapamycin, an mTOR inhibitor, prevented the Fen-mediated increase and Ang II-mediated decrease in LC3-II protein expression. α-tubulin is used for loading control. Rapa, rapamycin. Fen, fenoldopam; Ang II, angiotensin II. *n* = 4/Group, *** *p* < 0.001, ns, not significant. One-way ANOVA, Bonferroni multiple comparison test.

Cyclic AMP activates either PKA or alternative exchange protein directly activated by cAMP (EPAC) [33,34], both of which lead to the phosphorylation and inhibition of the mammalian target of rapamycin (mTOR) [33,34]. This sequence of events results in the inhibition of mTOR which increases autophagy [35,36]. To determine whether Fen- and Ang II-regulated autophagy involves the mTOR signaling pathway, rapamycin, an mTOR inhibitor [36,37] was added to the cells treated with Fen or Ang II. Rapamycin prevented the Fen-mediated increase (Figure 7A) and Ang II-mediated decrease (Figure 7B) in LC3-II protein expression, indicating that the Fen-mediated increase and Ang II-mediated decrease in autophagy were mTOR-dependent.

Phosphorylated 70/ribosomal S6 kinase (P70S6K), a direct downstream target of mTOR, is commonly used in the evaluation of mTOR activity [36,37,38]. Therefore, we investigated the effect of Fen and Ang II on the expression of P70S6K, using the mTOR/P70S6K inhibitor, rapamycin [35,37] (Supplementary Figure S10). Fen decreased (Supplementary Figure S10A) whereas Ang II increased (Supplementary Figure S10B) P70S6K levels, both of which were prevented by Rp-cAMPS, a PKA inhibitor (Supplementary Figure S10A,B). These indicate that the stimulatory and inhibitory effects of autophagy by Fen or Ang II, respectively, were dependent on the mTOR/P70S6K signaling pathway.

### 2.7. Proliferation of VSMCs Is Regulated by Autophagy and cAMP Signaling

**Figure 8:**
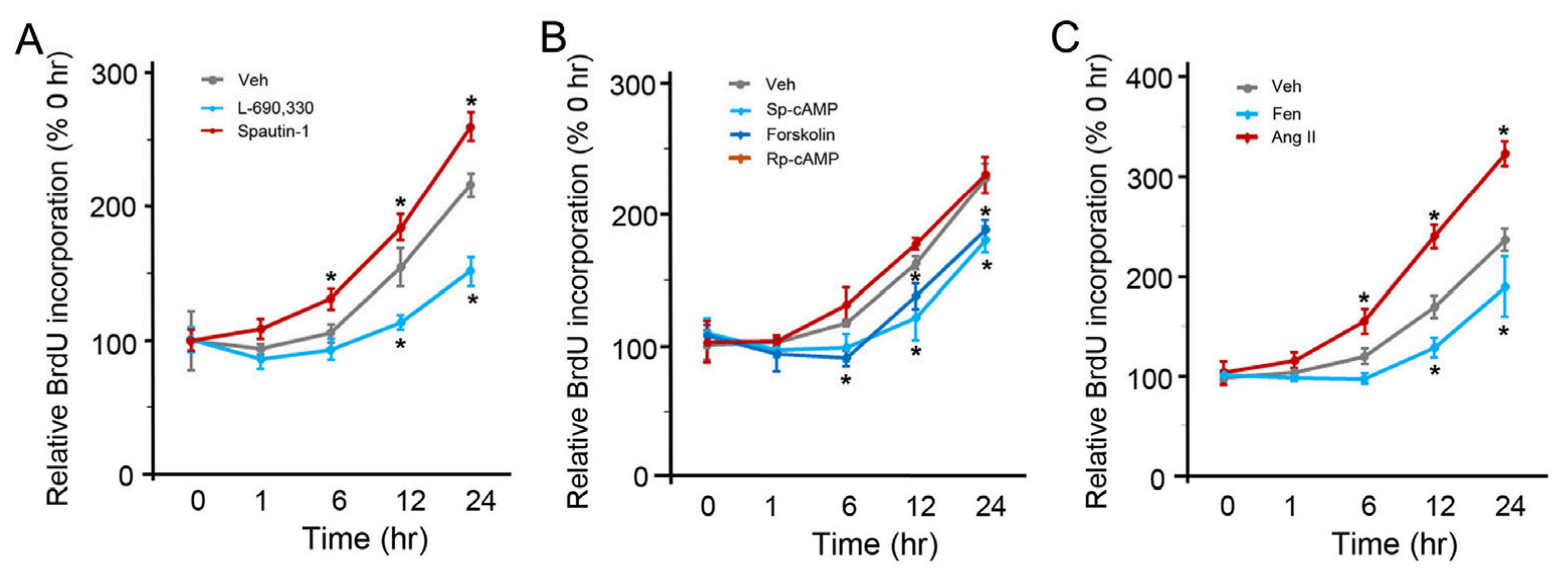
**Effects of autophagy and cAMP signaling modulators on VSMC proliferation measured by BrdU incorporation assay**. (**A**) VSMCs were treated with vehicle (Veh), L-690,330, an autophagy inducer (10 µmol/L), or spautin-1, an autophagy inhibitor (10 µmol/L), at the indicated times. (**B**) VSMCs were treated with vehicle (Veh), Sp-cAMPS, a PKA activator (1.0 µmol/L), forskolin, an adenylyl cyclase activator (10 µmol/L), or Rp-cAMPS, a PKA inhibitor (50 µmol/L), at the indicated times. (**C**) VSMCs were treated with vehicle (Veh), Fen (1 µmol/L), or Ang II (100 nmol/L) at the indicated times. Values (100% at 0 time) are shown, using Veh-treated cells at their initial time point (0 h) for normalization. (**A**–**C**) Veh, vehicle; Fen, fenoldopam; Ang, Ang II; BrdU, 5-bromo-2-deoxyuridine. Data are mean ± standard deviation. *n* = 3/Group, * *p* < 0.05 vs. Veh. Two-way ANOVA, Bonferroni multiple comparison test.

We next delved into whether autophagy, as related to Fen and Ang, affects VSMC proliferation, a pathophysiological process that is critical in the pathogenesis of vascular disorders [2,15,39,40]. The effect of autophagy on VSMC proliferation has been studied using autophagy inducers and repressors. L-690,330, an autophagy inducer [41], decreased, whereas spautin-1, an autophagy repressor [13], increased the quantity of VSMCs, determined by cell counting (Supplementary Figure S11A), MTT assay (Supplementary Figure S11A), and BrdU incorporation (Figure 8A).

Either the PKA stimulator, Sp-cAMPS [13,32], or the adenylyl cyclase activator, forskolin, decreased basal VSMC count (Supplementary Figure S11B), MTT assay (Supplementary Figure S11B), and BrdU incorporation (Figure 8B). By contrast, the PKA inhibitor, Rp-cAMPS [13], by itself, had no significant effect on basal VSMC count (Supplementary Figure S11B), MTT assay (Supplementary Figure S11B), and BrdU incorporation (Figure 8B).

However, as expected, Fen, which increases adenylyl cyclase activity, decreased VSMC count (Supplementary Figure S11C), MTT assay (Supplementary Figure S11C), and BrdU incorporation (Figure 8C), whereas Ang II, which decreases adenylyl cyclase activity, increased VSMC count (Supplementary Figure S11C), MTT assay (Supplementary Figure S11C), and BrdU incorporation (Figure 8C). These findings are consistent with their effects on intracellular cAMP production (Figure 4A,B), indicating that Fen and Ang II regulate autophagy and VSMC proliferation in a cAMP-dependent manner.

### 2.8. AT_1_R-Mediated VSMC Proliferation Is Attenuated by D1-like Receptors

**Figure 9:**
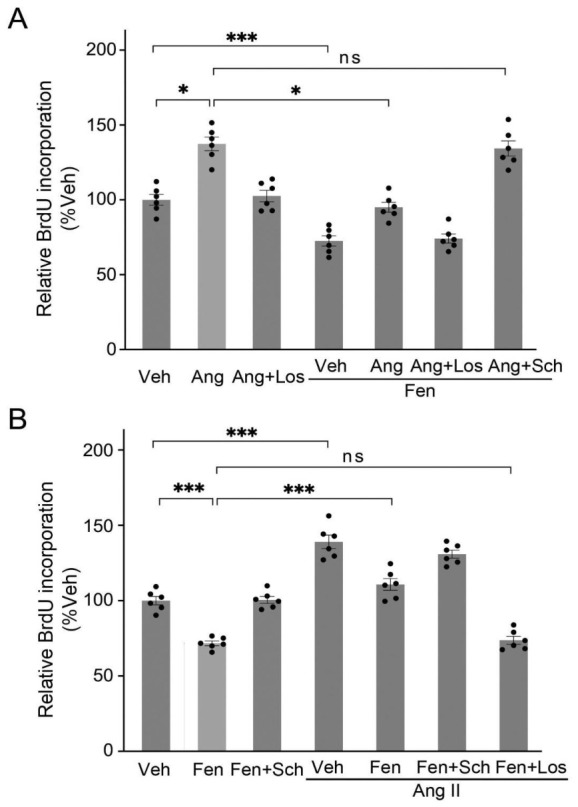
**Counter-regulatory effects of AT_1_R and D1-like receptors on VSMC proliferation**. (**A**) VSMCs were treated with Ang II (Ang,100 nmol/L), Fen (1 µmol/L) without or with Los (Los, 10 µmol/L), Sch (1 µmol/L) or their combination, as indicated, for 6 h. Fen attenuated the Ang II-induced cell proliferation in VSMCs. Los (AT_1_R antagonist) prevented the ability of Ang to counteract the ability of Fen to decrease cell proliferation, whereas the addition of Sch (D1-like receptor antagonist) restored the ability of Ang II to increase cell proliferation. (**B**) VSMCs were treated with Fen (1 µmol/L), Ang II (100 nmol/L) without or with Sch (1 µmol/L), and Los (10 µmol/L), as indicated, for 6 h. Ang II attenuated the Fen-mediated-decrease in cell proliferation. Sch restored the ability of Ang II to increase cell proliferation. By contrast, Los (Los, 10 µmol/L) restored the ability of Fen to decrease cell proliferation. Cell proliferation was quantified by BrdU incorporation, as described in the Section 4. Relative proliferation (%) is shown using the Veh group for normalization. (**A**,**B**) Veh, vehicle; Fen, fenoldopam; Sch, Sch23390; Ang or Ang II, angiotensin II; Los, losartan; BrdU, 5-bromo-2-deoxyuridine. *n* = 6/Group, * *p* < 0.05, *** *p* < 0.001, ns, not significant. One-way ANOVA, Bonferroni multiple comparison test.

As assessed by cell counting (Supplementary Figure S11), MTT assay (Supplementary Figure S11), and BrdU incorporation (Figure 8), Ang II (100 nmol/L, 6 h) stimulated VSMC proliferation. The Ang II-mediated stimulation of VSMC proliferation, measured using BrdU incorporation, was prevented by pre-treatment with the AT_1_R antagonist, losartan (Figure 9A), or the D1-like receptor agonist, Fen (Figure 9A). Furthermore, the inhibitory effect of Fen on the Ang II-mediated increase in VSMC proliferation was prevented by the D1-like receptor antagonist, Sch23390 (Figure 9A). These results align with their effects on intracellular cAMP production, i.e., Ang II decreases (Figure 4B and Supplementary Figure S8B) whereas Fen increases cAMP production (Figure 4A and Supplementary Figure S8A).

### 2.9. D1-like Receptor-Mediated Decrease in VSMC Proliferation Is Prevented by AT_1_R

The Fen-mediated inhibition of VSMC proliferation, measured by BrdU incorporation, was prevented by pre-treatment with the D1-like receptor antagonist, Sch23390 (Figure 9B), or Ang II, the endogenous AT_1_R agonist (Figure 9B), which was blocked by the AT_1_R antagonist, losartan (Figure 9B). The Ang II-mediated stimulation of VSMC proliferation, also measured by BrdU incorporation, was prevented by pre-treatment with the AT_1_R antagonist, losartan. The blockage of Ang II on Fen--mediated decrease in VSMC proliferation was prevented by AT_1_R antagonist, losartan (Figure 9B). These correspond to their effects on intracellular cAMP production, i.e., Fen stimulates whereas Ang II inhibits cAMP production (Figure 4 and Supplementary Figure S8).

Therefore, our results demonstrate that D1-like receptors and AT_1_Rs counter-regulate each other’s effects on cAMP production, autophagy, and VSMC proliferation.

## 3. Discussion

Autophagy operates as an essential cellular quality-control mechanism that removes and recycles dysfunctional intracellular macromolecules and organelles [2,13,14,15,16,17,18,19,25,27,33,34,35,36,38,40,41]. In this study, we demonstrated that autophagy in VSMCs is regulated by G protein-coupled receptors (GPCRs), specifically D1-like receptors and AT_1_R. We found that D1-like receptors increased autophagy by stimulating the cAMP pathway whereas AT_1_R decreased autophagy by inhibiting the cAMP pathway. Accordingly, these results indicate that D1-like receptors and AT_1_R counter-regulate cAMP production, autophagy, and proliferation in VSMCs.

The role of D1-like receptors in the renal-mediated regulation of sodium excretion and blood pressure is well-known [3,4,5,6,7,26,42,43], but their role in autophagy is not completely understood. In the kidney, Fen, a D1-like receptor agonist, increases the protein expression of hallmark proteins in the autophagic process [13]. Our current study showed that the activation of D1-like receptors increased the protein expression of LC3-II, which was augmented by treatment with chloroquine, an inhibitor of autophagosome-lysosome fusion. These results show that, in VSMCs, D1-like receptors induce autophagy rather than block the lysosomal degradation system. The D1-like receptor-mediated induction of autophagy is cAMP-dependent, which concurs with a report that D_1_R activation, through cAMP signaling, increases the autophagic degradation of NLRP3 inflammasomes in macrophages [14]. The dependence of the D1-like receptor-mediated induction of autophagy on cAMP signaling agrees with the fact that D1-like receptors couple with Gαs and increase cAMP levels [3,4,5,6,7,13,14,22]. It is also reported that the D2-like receptors, D_3_R, and D_4_R also stimulate autophagy [36,44], but the mechanisms involved are most likely different from those in D1-like receptors. Our results show that the D1-like receptor-mediated increase in autophagy is cAMP-dependent [13,14]. The cAMP-mediated inhibition of the mTOR signaling leads to an increase in autophagy [33,34]. In HeLa or HEK293 cells stably overexpressing *DRD1*, *DRD3*, and *DRD5*, D_2_R and D_3_R stimulated whereas D_1_R and D_5_R inhibited autophagy [45]. The discrepancy between this overexpression study on the inhibitory effect of D_1_R on autophagy [45] and the stimulatory effect of D_1_R on autophagy in our study, which is supported by other studies [14,46], as well as the observed impaired ability of the D1-like receptor agonist, SKF38393, to stimulate LC3 II protein expression in the striatum of *Drd1* knockout mice [46], remains to be clarified. Nevertheless, the majority of studies have shown that the cAMP pathway stimulates autophagy (*vide infra*).

Our studies show that Fen, as a robust driver of genuine autophagic flux (Figure 1D), alone increases the protein expression of LC3-II and therefore autophagy, which is associated with a decrease in mTOR activity, measured by a decrease in the phosphorylation of P70S6K (Supplementary Figure S10). Rapamycin, an mTOR inhibitor, did not increase the LC3-II protein expression at the basal level (Figure 7A,B), which is consistent with previous reports [47,48], but rather prevented the Fen-mediated increase in LC3-II protein expression (Figure 7A). Rapamycin, an allosteric inhibitor of mTOR, acts as a road blocker or bottleneck that structurally alters mTOR signaling and physically prevents downstream signaling cascades [37]. Once this pathway is disrupted, parallel signaling pathways become prominently engaged [49]. The AT_1_R counter regulatory mechanism could be initiated to suppress autophagy at the cAMP (Figure 4) and adenylyl cyclase levels. Although fenoldopam alone suppresses the baseline proliferative Akt/mTOR pathway in VSMCs [8], allosteric rapamycin-mediated decrease in P70S6K activity removes downstream negative feedback constraints, which drives a compensatory hyper-activation of upstream PI3K/Akt signaling [50,51]. The increased PI3K/Akt signaling increases downstream mTOR activity [52] and decreases ULK1 activity by phosphorylation of ULK1 at its inhibitory site at Ser757 [53]. Concurrently, fenoldopam-mediated increase in cAMP/PKA signaling is forced to phosphorylate AMP-activated protein kinase (AMPK) due to the block of mTOR signaling. PKA is well-documented to phosphorylate the α-subunit of AMPK at inhibitory Ser485/491 residues, suppressing alternative canonical rescue switches [54,55]. The EPAC could be forced to be activated, which can, either through PI3K/Akt or direct phosphorylation of AMPK at inhibitory residues Ser173/Ser485, inhibit AMPK [56]. The confluence of allosteric disruption, compensatory Akt hyper-activation, and concurrent PKA-mediated AMPK inhibition caps further autophagosome generation, which can explain the apparent paradox observed in the combination treatment group (Figure 7). The discussed PI3K/Akt, AMPK, and EPAC pathways, along with mitogen-activated protein kinase/extracellular signal-regulated kinase, redox signaling, and mTORC2, warrant further investigation.

There is considerable debate regarding the extent to which AT_1_Rs regulate autophagy. In VSMCs, Ang II can induce cellular hypertrophy [57] and proliferation [58] through an increase in autophagy. Some studies have shown that the Ang II-mediated increase in autophagy could be caused by another Ang II receptor, i.e., AT_2_R, which generally opposes the effects of AT_1_R on cellular signaling, vascular tone, oxidative stress, cell growth, autophagy, apoptosis, and inflammation [59,60]. It is possible that AT_2_R cross-talks with both AT_1_R and D1-like receptors in order to regulate autophagy and cell proliferation in VSMCs [61,62,63]. However, Ang II has also been reported to decrease autophagy in VSMCs [15] and other cell types involved in diverse pathologies. For example, Ang II suppresses the expression of autophagy-related proteins in mouse VSMCs, which plays an important part in the progression of abdominal aortic aneurysm [64]. In mouse skeletal muscles, Ang II attenuates autophagy in vivo by decreasing the conversion of LC3B-I to LC3B-II, increasing p62/SQSTM1 protein expression, and decreasing the number of autophagic vacuoles [25]. In endothelial cells, Ang II also decreases autophagy, which orchestrates an endothelial-to-mesenchymal transition [65]. In VSMCs, skeletal muscle cells, cardio-fibroblasts, and cardiomyocytes, AT_1_R activation increases p62/SQSTM1 protein expression, showing an inverse correlation with LC3-II protein expression [25,66,67]. Thus, Ang II can also decrease autophagy [15,25,40,64,65,66,67,68]. Indeed, in our study, using rat thoracic VSMCs, AT_1_R decreased autophagy in a time- and concentration-dependent manner, which is consistent with its ability to increase VSMC proliferation. As with the discrepant effects of rapamycin on autophagy, the discrepant effects of Ang II on autophagy could also be related to the use of different Ang II concentrations, cell types, functional status of the cells, signaling compartmentalization, and microenvironment [15,16,17,25,28,31,40,57,58,64,65,66,67,68], underscoring the need for additional studies.

AT_1_R activation has been observed to decrease autophagy and increase cell proliferation of different cell populations, including VSMCs [2]. In addition to the activation of Gαq/11 and Gα12/13 signaling [9], AT_1_R is also able to directly or indirectly activate Gαi and inhibit adenylyl cyclase activity, culminating in a decrease in cAMP production [10,29]. cAMP-PKA suppresses the Ang II-induced increase in VSMC proliferation and hypertrophy, which are important in vascular remodeling under hypertensive conditions [69]. AT_1_R negatively interacts with both D_1_R and D_5_R [5,20,21,22], potentially resembling a similarity with how the heterodimerization between AT_1_R and α_2c_-adrenergic receptor affects cAMP signaling [70]. Our data demonstrated that the AT_1_R-mediated decrease in cAMP production and autophagy was prevented by treatment with losartan (Figure 2C) and Rp-cAMPS (Figure 4B), which seems to contradict the PKA activity assay data (Supplementary Figure S9), but it actually indicates that an Ang II-mediated decrease in autophagy did not proceed via a simple linear reduction in cAMP-PKA signaling, but rather a functional signaling multi-branched network. In addition to PKA, EPAC is an alternative effect of cAMP [33,34]. EPAC and PKA often synergistically or oppositely regulate the same biological process [56]. Our data further suggested that Ang II-mediated suppression of autophagy in VSMCs is dependent on a highly coordinated, non-linear cross-regulation among the cAMP-PKA, cAMP-EPAC, and AMPK signaling networks, and AT_1_R and D1-like receptors counter-interacting at the cAMP (Figure 4) and adenylyl cyclase levels. Exposure to Ang II significantly reduced intracellular cAMP levels (Supplementary Figure S8), decreased LC3-II expression (Figure 2C and Figure 3B), and hyper-activated phospho-P70S6K (Supplementary Figure S10B). This suppressive effect on autophagosome accumulation was completely blocked by co-treatment with the competitive PKA inhibitor Rp-cAMPS, which returned both LC3-II and p-P70S6K expression back to baseline/vehicle levels (Figure 3B). The pronounced decrease in PKA activity with the combination of Rp-cAMPS and Ang II (Supplementary Figure S9) could disinhibit AMPK by removing PKA-mediated inhibitory phosphorylation at Ser485/491 [54,71,72], which can potentially cooperate with the EPAC shunts [33], leading to inhibition of mTOR and increase in autophagy. In addition to the parallel downstream AMPK and EPAC activation, compensatory hyper-activation of G-protein-coupled receptor kinases, and β-arrestin mobilization, heterologous desensitization and subsequent endocytic uncoupling of the surface AT_1_R [73,74] could also contribute to the restoration of mTOR activity and LC3 protein expression back to the baseline/vehicle level. Phosphorylation of mTORC1/C2, AMPK, Akt components, and protein phosphatase 2 following AT_1_R and D1-like receptor agonists and antagonists will be determined by these specific phospho-serine and phospho-threonine antibodies in the future. Nevertheless, our study shows that cAMP signaling is the common pathway by which D1-like receptors and AT_1_R counter-regulate autophagy in VSMCs.

In addition to the aforementioned cross-regulated network downstream cAMP signaling, it is important to note that autophagy and cell proliferation are also cross-regulated by multiple pathways. Specifically, the activation of AT_1_R and dopamine D1-like receptors can induce the formation of inositol 1,4,5-triphosphate (IP3), subsequently increasing intracellular Ca^2+^ levels to modulate (increase and/or decrease) the autophagic flux in VSMCs [9,75,76]. Future studies are needed to clarify the potential crosstalk of cAMP signaling with IP3- Ca^2+^ signaling, related PI3K signaling [77] and other pathways in the regulation of autophagy by these two GPCRs in VSMCs.

Autophagy is progressively being recognized as a critical determinant of the VSMC phenotype, where it prevents the VSMC switch from a contractile phenotype to synthetic phenotype and consequently inhibits VSMC proliferation [1,2]. Both rapamycin-like and non-rapamycin-like autophagy inducers have been reported to diminish VSMC proliferation [35,78]. Interestingly, paeonol, an active component of the root bark of Moutan Cortex, upregulates autophagy and decreases VSMC proliferation [35]. Caffeine, a widely consumed CNS stimulant worldwide, also reduces VSMC proliferation by stimulating autophagy in vitro and in vivo [38]. These studies show that autophagy decreases VSMC proliferation and reinforces not only our findings, but also other reports, showing that the inhibition of autophagy increases VSMC proliferation [79]. However, there are also other studies showing that excessive autophagy causes vascular dysfunction and hypertension [80], and the inhibition of autophagy can mitigate hypoxia-induced proliferation of VSMCs [81]. It has been suggested that the degree of autophagy may determine whether autophagy is beneficial or detrimental [82,83].

The Fen-mediated decrease and Ang II-mediated increase in cell proliferation and their potential roles in migration, synthesis of extracellular matrix, and calcium signaling in VSMCs, in coordination with the endothelial production of nitric oxide, prostaglandins, and lipoxygenases, play critical roles in vascular remodeling, vascular tone, and blood pressure regulation [2,84]. Therefore, the finding in our study regarding the counter-regulation of autophagy and proliferation in VSMCs by D1-like receptors and AT_1_R has clinical significance. In healthcare settings, the intravenous administration of Fen is used to decrease blood pressure during episodes of severe hypertension [85]. AT_1_R blockers or angiotensin-converting enzyme inhibitors (ACEis) are therapies for hypertension [85,86]. The combination of Fen and enalapril, an ACEi, causes a greater and more sustained reduction in blood pressure levels than when either drug is used alone [87]. The use of selective D_1_R or D_5_R agonists with AT_1_R blockers or ACEi and autophagy inducers, such as spermidine [88] or trehalose [89] to treat hypertension, merits investigation to guide future interventions against drug-resistant hypertension in humans.

Although the A10 cell line serves as a robust and genetically stable model for evaluating these shared signaling pathways between AT_1_R and D1-like receptors, the use of cell lines is a limitation that should be considered due to its embryonic origin with higher proliferation and synthetic phenotype than mature adult VSMCs. The results obtained in our study are specific to this rat embryonic VSMC cell line; therefore, future endeavors will be required to validate these findings in primary cultured rodent and human VSMCs. These cells were cultured in low-serum conditions, which may not adequately replicate the in vivo diversity of VSMCs in complex microenvironments. Additionally, the results of this study cannot be generalized because there are variations in D_1_R, D_5_R, and AT_1_R protein expressions at different stages of life. Although we measured the basal level of protein expression of D_1_R, D_5_R, and AT_1_R, the closely related receptors, such as D2-like receptors, AT_2_R, and Mas receptors, were not characterized. Their potential crosstalk with AT_1_R and D1-like receptor subtypes on autophagy regulation in health and disorders, such as inflammation, hypertension, and atherosclerosis, can affect receptor expression, signaling, and function. Endothelial cells and VSMCs coordinately regulate vascular tone. Dysfunction of both cell types are participated in the vascular remodeling and pathogenesis of hypertension. consequently, the interaction of AT_1_R and D1-like receptors, particularly the nitric oxide production in the endothelial cells will be investigated in the future. To untangle the systemic and cell-specific contribution of this crosstalk, future endeavors should also evaluate these pathways in vivo through chronic controlled micro-infusion of selective pharmacological agonists, antagonists, inhibitors, and activators in their gene-manipulated global, VSMC-specific *Agtr1a* and/or *Drd1*/*Drd5* knockout or transgenic mice and/or humanized *AGTR1* and *DRD1*/*DRD5* transgenic mice and their wild-type littermates to these targeted regimens. We hope to map out the interplay of these two GPCRs on the regulation of vascular tone, remodeling, and blood pressure.

## 4. Materials and Methods

### 4.1. Antibodies and Reagents

Anti-LC3-II (Cat. No. 3868), anti-beclin-1 (BECN1) (Cat. No. 3495), anti-P70S6K (Cat. No. 9202), anti-phospho-P70S6K (Cat. No. 9205), and phospho-PKA substrate (RRXS*/T*, Cat. No. 9624) antibodies were purchased from Cell Signaling Technology (Danvers, MA, USA). Anti-D_1_R (BioMatik, Kitchener, ON, Canada) and anti-D_5_R antibodies were made in-house and their specificities have been previously verified [42,90]. Anti-AT_1_R (Cat. No. GTX89149) antibodies were purchased from GeneTex (San Antonio, TX, USA). Anti-α-tubulin (Cat. No. ab4074) and anti-GAPDH (Cat. No. ab8245) antibodies for loading controls were purchased from Abcam (Cambridge, MA, USA). Anti-β-actin antibodies (Cat. No. A1978) were purchased from MilliporeSigma (Burlington, MA, USA). The dilutions of the primary antibodies are listed in the Supplementary Table S1. L-690,330, an inositol monophosphatase inhibitor, was purchased from Novus Biologicals (Pittsburgh, PA, USA). Spautin-1, an autophagy inhibitor, was purchased from Griffin Biotech (Hong Kong, China). Culture media and fetal bovine serum (FBS) were purchased from Invitrogen (Gaithersburg, MD, USA). Fenoldopam, Sch23390, angiotensin II, losartan, Rp-cAMPS, Sp-cAMPS, forskolin, chloroquine, rapamycin, and other reagents were purchased from Sigma-Aldrich (St. Louis, MO, USA). Stock solutions of the reagents were prepared in phosphate-buffered saline (PBS), except for L-690,330, Spautin-1, forskolin, rapamycin, and Sch23390, which were prepared in dimethyl sulfoxide (DMSO).

### 4.2. Cell Culture, siRNA, and Transfection

Embryonic thoracic VSMCs, from normotensive Berlin–Druckrey C rats (CRL-1476, ATCC, Manassas, VA, USA) were cultured, as previously described [32]. We used aortic smooth muscle cells because angiotensin II [91,92] and dopamine [32,93] participate in the regulation of VSMC proliferation or conduit artery stiffness. VSMCs were cultured in Dulbecco’s Modified Eagle’s Medium (DMEM) with10% FBS and 2 mM pyruvate and incubated at 37 °C in 5% CO_2_/95% humidified air.

Specific *Drd1* (5′-ggtccaaggtgaccaacttct-3′, gene ID: 24316), *Drd5* (5′-gccaagatgaccaacatctt-3′, Gene ID: 25195), *Agtr1a* (5′-cagcttggtggtgattgtcat-3′, gene ID: 24180), and scrambled (5′-gggacgcactacctagacttt-3′) siRNAs (Invitrogen) were transfected into VSMCs and grown in 12- or 24-well plates, using Lipofectamine 2000 transfection reagent (Invitrogen) [94]. Twenty nM unmodified, double-stranded siRNAs were diluted in Opti-MEM medium, gently mixed, adjusted to a one-to-three ratio, and incubated with diluted transfection reagent for 15 min. The transfection complexes were drop-wised into 6-well plates.

The efficiency of siRNA silencing was determined by quantitative real-time qRT-PCR (Supplementary Figure S12A) and immunoblotting (Supplementary Figure S12B).

Total RNA, extracted from VSMCs using TRIzol (Invitrogen, Frederick, MD, USA) and re-extracted using NucleoSpin RNA kit (Takara Bio USA, San Jose, CA, USA), was reverse transcribed using GoScript reverse transcriptase (Promega, Madison, WI, USA). The real-time qRT-PCR reaction, previously described [95], used the following primers: *Drd1*, forward primer 5′-gagtggttgggggaagtctg-3′; reverse primer 5′-aggtgtcgaaaccggatgac-3′; *Drd5*, forward primer 5′-gtatcatcagcgtggaccgt-3′; reverse primer 5′-tccgtcctcccttctagctc-3′; *Agtr1a*, forward primer 5′-ttcgtggcttgagtcctgtt-3′; reverse primer 5′-cgaaatccacttgacctggtg-3′; and *Gapdh*, forward primer 5′-tgttctagagacagccgcatc-3′; reverse primer 5′-ctcgctcctggaagatggtg-3′. Gene expression was calculated using the 2^−ΔΔCt^ method.

The VSMCs that were ~75–80% confluent were partially serum-starved overnight by culturing them in DMEM with 1.5% FBS. These cells positively stained for smooth muscle-specific actin (MilliporeSigma, CBL171) and exhibited the typical hill-and-valley morphology. VSMCs within passages 6–10 and negative for Mycoplasma, as confirmed by a universal Mycoplasma detection kit (ATCC 30-1012K), were used throughout the study. The number of cells was counted on a hemocytometer under an inverted microscope.

### 4.3. Western Blotting

Western blotting was performed to quantify protein expression levels. The cell lysates were adjusted to the same protein concentration. The extracted protein samples, separated by sodium dodecyl sulfate-polyacrylamide gel electrophoresis (SDS-PAGE), were transferred onto a nitrocellulose membrane and probed with primary antibodies and appropriate horseradish peroxidase-conjugated secondary antibodies. The protein bands on the membranes were visualized using chemiluminescence imaging.

### 4.4. Autophagy Evaluation

Autophagic activity was measured by immunoblotting for LC3-II and BECN1, as previously described [96,97]. Their specificities were confirmed using *LC3B* knockout and *BECN1* siRNA cells (Cell Signaling Technology). The densities of the LC3-II and BECN1 protein bands, normalized by α-tubulin, β-actin, or GAPDH density, were measured in lysates of cells treated with vehicle or chloroquine (10 μmol/L), an autophagy inhibitor [13,24], during the final 2 h of a 6 h stimulation with either Fen (1 μmol/L) or Ang II (100 nmol/L), as indicated in the individual experiments. The cell lysates were then electrophoresed, and the extracted protein samples were separated by SDS-PAGE.

LC3-II puncta were observed under a confocal immunofluorescence microscope, as previously described [13]. Briefly, VSMCs, cultured on glass coverslips, were fixed with 4% formaldehyde in PBS for 15 min and permeabilized with 0.25% Triton X-100 for 5 min at room temperature. The permeabilized cells were incubated in PBS, supplemented with 0.05% Tween 20 and 2% BSA for 60 min and incubated with primary anti-LC3-II antibody overnight at 4 °C. After the primary antibody labeling, the VSMCs were incubated with Alexa Fluor 488-conjugated anti-rabbit IgG, and the nuclei were stained with DAPI (4′-6-diamidino-2-phenylindole). The images of the stained cells were visualized using a ZEISS microscope and analyzed with ImageJ (Version 1.54d) (NIH, Bethesda, MD, USA).

### 4.5. Cyclic Adenosine Monophosphate (cAMP) Measurement

The concentration of cAMP was measured by an immunoassay kit (BioVision, Milpitas, CA, USA). The VMSCs were cultured in 12-well plates with complete culture medium until they reached 85% confluence. Afterward, the VMSCs were serum-starved and pretreated with 3-isobutyl-1-methylxanthine (IBMX) (MilliporeSigma Aldrich), a non-selective phosphodiesterase inhibitor, for 30 min prior to treatment with D1-like receptor and/or AT_1_R agonists and/or antagonists at 37 °C for 15 min. Forskolin (10^−5^ mol/L) was added to the VSMC suspensions 10 min before being treated with Ang II (10^−10^–10^−6^ mol/L) for 15 min. The protein concentrations of the samples, measured by the Pierce BCA Protein Assay Kit, were used to normalize cAMP levels. Additionally, the data were normalized in reference to the control (0 mol/L, vehicle phosphate-buffered saline [PBS], or 0.01% dimethyl sulfoxide [DMSO]) group. Each experimental treatment was performed three times in order to ensure consistency and reliability.

### 4.6. MTT Assay

The actively dividing cells exhibited a high rate of reduction of 3-(4, 5-dimethylthiazol-2-yl)-2, 5-diphenyltetrazolium bromide (MTT), which was measured by an MTT assay kit (Cayman Chemical, Ann Arbor, MI, USA). VSMCs (5 × 10^3^) in 100 µL of culture medium were loaded into each well of a 96-well plate and placed in a 5% CO_2_ incubator for 48 h. The cells in the CO_2_ incubator were treated with selected drugs for 0, 1, 6, 12, and 24 h before the end of the incubation period. At the end of the incubation period in the drug-treated or vehicle-treated VSMCs, MTT solution (10 µL) was added to the complete culture medium (final concentration, 4 mg/mL), gently shaken for one minute, and incubated at 37 °C for 4 h. The formazan products were dissolved in DMSO, and their optical densities were spectrophotometrically measured at 570 nm in a microplate reader (Molecular Devices).

### 4.7. Bromodeoxyuridine (BrdU) Incorporation Assay for Cell Proliferation

The incorporation of BrdU into VSMCs was measured by a BrdU incorporation assay kit (Millipore Sigma, Burlington, MA, USA) [39]. The VSMCs were cultured for 24 h in a 96-well plate and pulse-labeled with BrdU (10 μmol/L in PBS, pH 7.4) at 37 °C. Then, the VSMCs were fixed with FixDenat solution for 30 min at room temperature. After removing the FixDenat solution, anti-BrdU monoclonal antibodies (100 μL/well) were added and incubated for 60 min. After washing the plates three times with washing buffer, the secondary antibodies, conjugated with horseradish peroxidase (100 μL/well), were added and the plates were incubated for 30 min at room temperature. Then, the plates were washed three times with PBS, and tetramethyl-benzidine (100 μL) was added into each well and incubated in the dark for 30 min at room temperature before measuring the absorbance at 450 nm.

### 4.8. Statistical Analysis

All of the results are presented as mean ± standard error of the mean (SEM), unless otherwise stated. Significant differences among three or more groups were determined by one-way or two-way ANOVA, followed by Bonferroni multiple comparison test or pairwise comparison test for each group, unless otherwise stated in the figure legends. *p* < 0.05 was considered statistically significant (Prism 9.5.1 version, Boston, MA, USA).

## 5. Conclusions

Under our experimental conditions in VSMCs, AT_1_R and dopamine D1-like receptors exert a reciprocal counter-regulation on autophagy and cell proliferation in VSMCs. Specifically, we demonstrated that this counter-regulatory axis is mTOR-dependent and mediated by intracellular cAMP signaling networks rather than a simple, linear G protein-cAMP-signaling cascade. This network interacts at multi-layered levels of AT_1_R and dopamine D1-like receptor signaling. This cAMP-dependent, mTOR-mediated counter-regulatory loop on the regulation of autophagy and cell proliferation provides a valuable insight into the therapies for hypertension, restenosis, and atherosclerosis by preventing VSMC proliferation and vascular remodeling.

## Data Availability

All data are presented within the article. Additional data available on request.

## Supplementary Materials

The following supporting information can be downloaded at: https://www.mdpi.com/article/10.3390/ijms27156784/s1.
