# Supplementary material for: Dopamine D1-like and Angiotensin II Type 1 Receptors Counter-Regulate Autophagy and Cell Proliferation in Rat Embryonic Thoracic Vascular Smooth Muscle Cells
Source: Int J Mol Sci. 2026 Jul 29;27(15):6784. doi: 10.3390/ijms27156784 (PMC13467237; doi:10.3390/ijms27156784)
Supplement: Supplementary file 1 [file ijms-27-06784-s001.zip › ijms-4301951-supplementary.pdf]

Supplementary file

Supplementary Figures

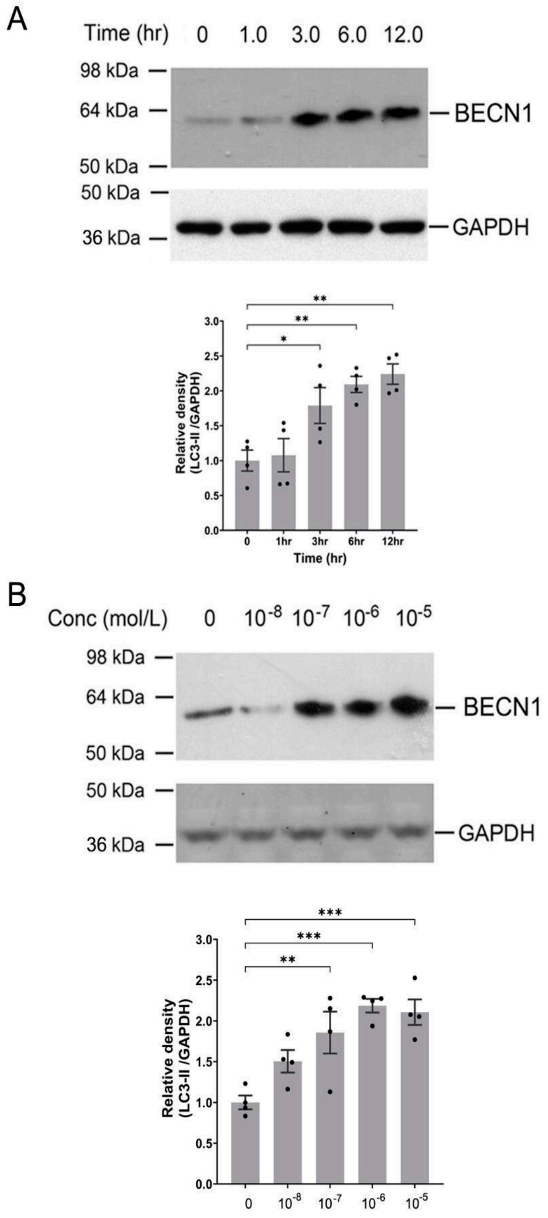

Supplementary Figure S1. Time course and concentration response of the fenoldopam (Fen)-mediated increase in beclin-1 (BECN1) protein expression in VSMCs. (**A**) VSMCs

were treated with Fen (1 μmol/L) at the indicated time. (**B**) VSMCs were treated with Fen for 6 hours by the indicated concentrations. (**A**,**B**) BECN1 protein expressions were quantified and expressed as the ratio of BECN1 over GAPDH densities, then normalized by their controls (0 hour or 0 mol/L). $n = 4$/Group, * $p < 0.05$, ** $p < 0.01$, *** $p < 0.001$ vs control (0 hour or 0 mol/L), ns, not significant. One-way ANOVA, Bonferroni multiple comparison test.

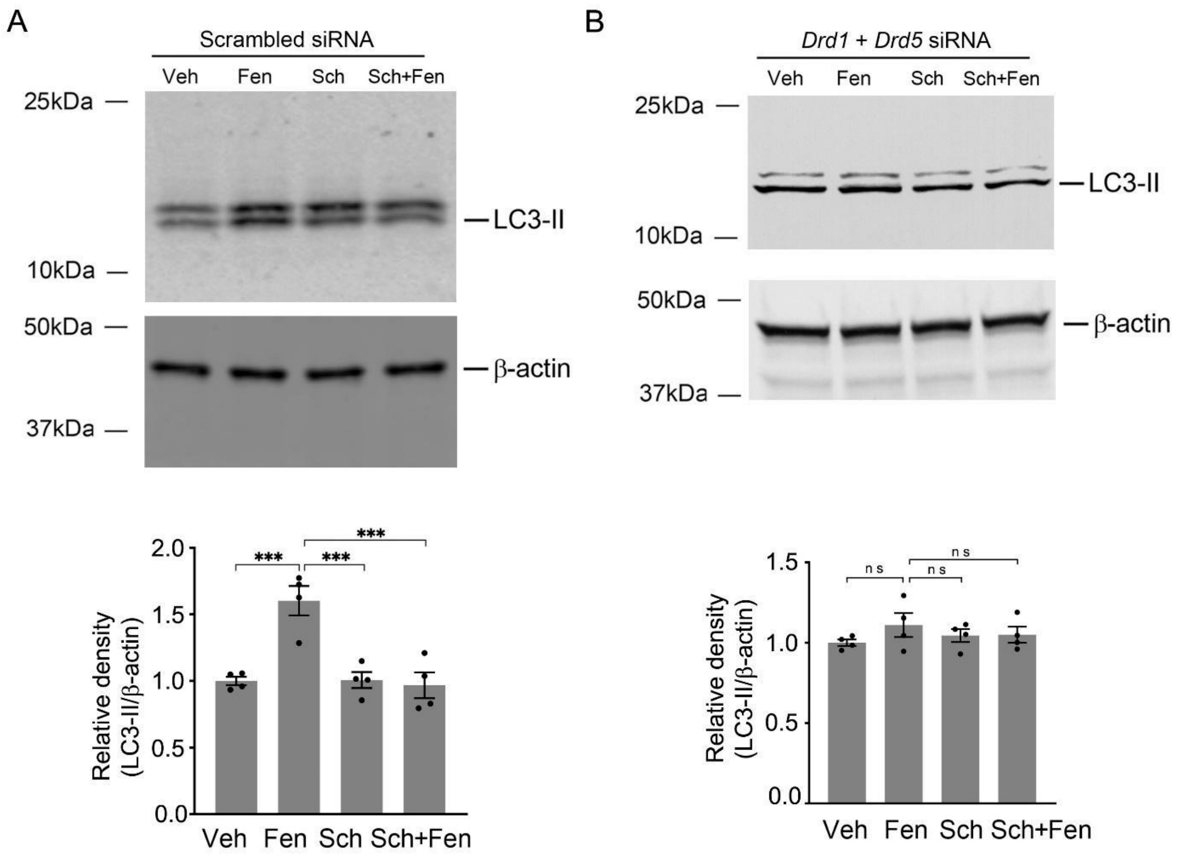

Supplementary Figure S2. LC3-II protein expression in VSMCs transfected with scrambled or combined *Drd1* and *Drd5* siRNAs. (**A**) VSMCs were transfected with scrambled siRNA (48 hours) and treated with vehicle (Veh) or Fen (1.0 μmol/L) for 6 hours, prior to the end of the 48 hour-transfection, in the absence or presence of the D1-like receptor antagonist, Sch23390 (Sch) (1.0 μmol/L, added 30 minutes prior to the addition of Fen). n = 4/Group, *** p < 0.001. One-way ANOVA, Bonferroni multiple comparison test. (**B**) VSMCs were transfected with combined *Drd1* and *Drd5* siRNAs (48 hours) and treated with Veh or Fen as in (A). Veh, vehicle; Fen, fenoldopam, Sch, Sch23390. n = 4/Group, ns, not significant. One-way ANOVA, Bonferroni multiple comparison test.

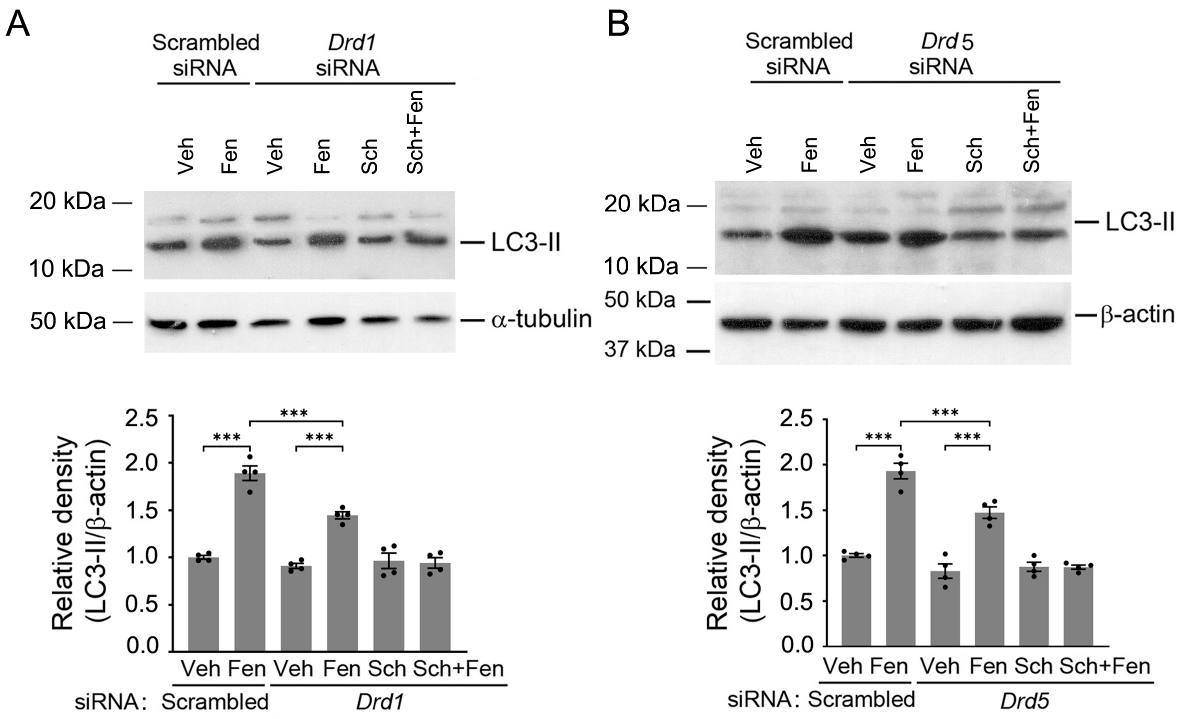

Supplementary Figure S3. LC3-II protein expression in VSMCs transfected with either *Drd1* or *Drd5* and their scrambled siRNAs. (**A**) VSMCs were transfected with *Drd1* or its scrambled siRNA (48 hours) and treated with vehicle (Veh) or Fen (1.0 µmol/L), as indicated, for 6 hours, prior to the end of the 48 hour-transfection, in the absence or presence of the D1-like receptor antagonist, Sch23390 (Sch) (1.0 µmol/L, added 30 minutes prior to the addition of Fen). (**B**) VSMCs were transfected with combined *Drd5* or its scrambled siRNA (48 hours) and treated with Veh or Fen as in (A). (**A**,**B**) Veh, vehicle; Fen, fenoldopam, Sch, Sch23390. n = 4/Group, *** $p < 0.001$, ns, not significant. One-way ANOVA, Bonferroni multiple comparison test.

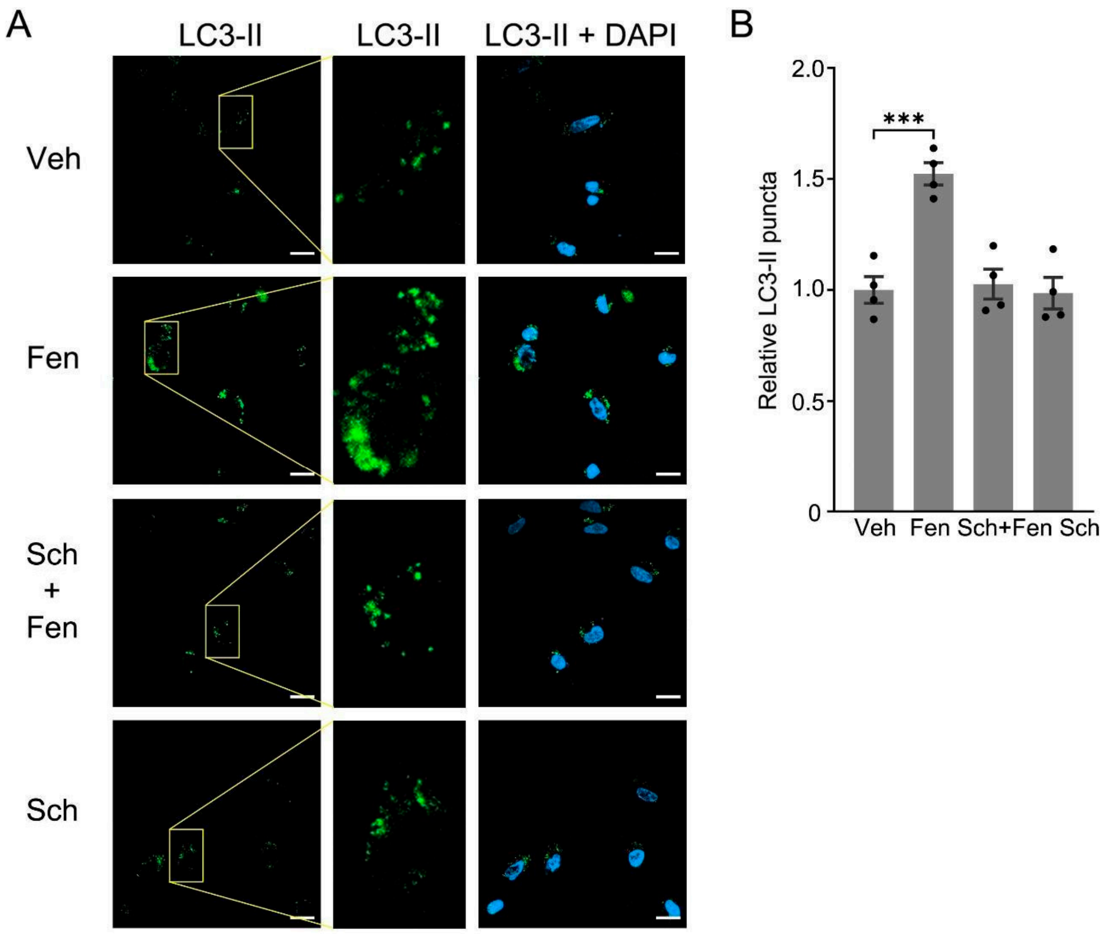

Supplementary Figure S4. Fen increases LC3-II puncta through D1-like receptors in VSMCs. (**A**) VSMCs were treated with Veh (vehicle), Fen (fenoldopam, 1.0 μmol/L), Sch (1 μmol/L) or Sch + Fen (combination of Fen and Sch) as indicated for 12 hours. Representative immunofluorescence images show endogenous LC3-II (green). Blue nuclei (DAPI). Scale bars, 10 μm. (**B**) The number of LC3-II puncta was counted by ImageJ; the number of cells were counted manually by their nuclei (cells on the edges were excluded for analysis). The number of LC3-II puncta was divided by the number of cells; each group was normalized by the average number of the Veh group. $n = 4$/Groups, *** $P < 0.001$. One-way ANOVA, Bonferroni multiple comparison test.

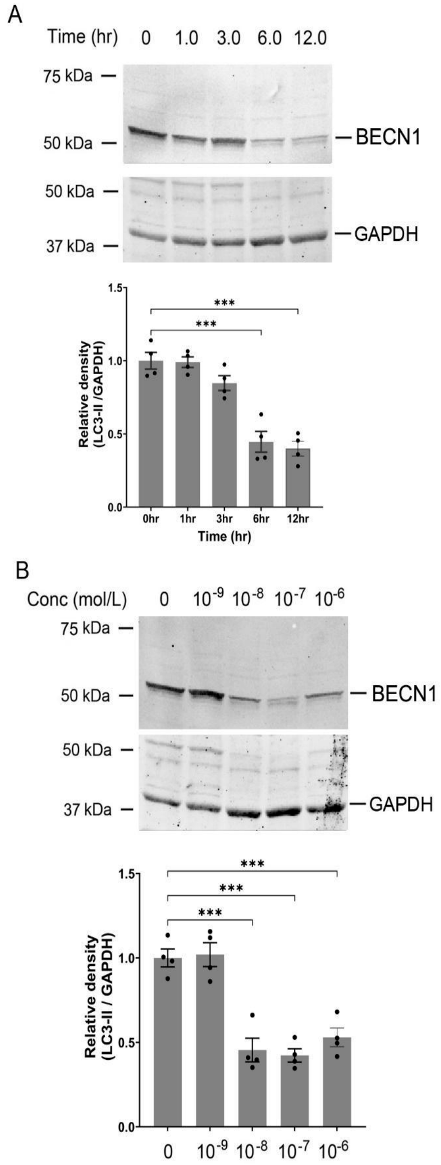

Supplementary Figure S5. Time course and concentration response of the Ang II-mediated decrease in beclin-1 (BECN1) protein expression in VSMCs. (**A**) VSMCs were treated with Ang II (100 nmol/L) at the indicated times. (**B**) VSMCs were treated with Ang II by the indicated concentrations for 6 hours. (**A**,**B**) BECN1 protein expression was quantified and expressed as the ratio of BECN1 over GAPDH densities, then normalized by the controls (0 hour or 0 mol/L). n = 4/Groups, *** $P < 0.001$. One-way ANOVA, Bonferroni multiple comparison test.

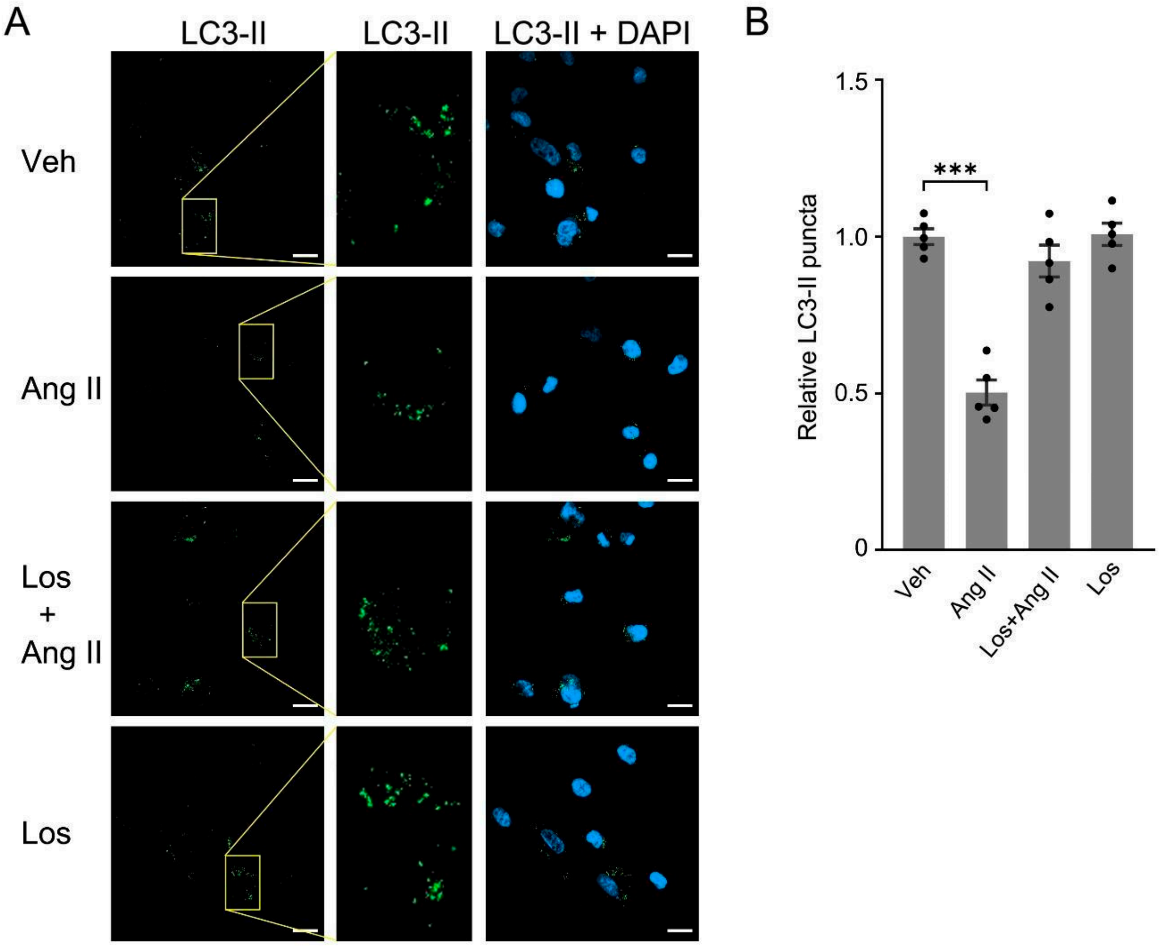

Supplementary Figure S6. Angiotensin II (Ang II) decreases LC3-II puncta through AT$_1$R in VSMCs. (**A**) VSMCs were treated with Veh (vehicle), Ang II (100 nmol/L), Los (losartan, 10 μmol/L), or their combination (Los + Ang II), as indicated for 12 hours. Representative immunofluorescence images show endogenous LC3-II (green). Blue, nuclei (DAPI). Scale bars, 10 μm. (**B**) The numbers of LC3-II puncta were counted by ImageJ; the number of cells were counted manually by their nuclei (cells on the edges were excluded for analysis). The number of LC3-II puncta were divided by the number of cells; each group was normalized by the average number of Veh group. n = 5/Group, *** $p < 0.001$. One-way ANOVA, Bonferroni multiple comparison test.

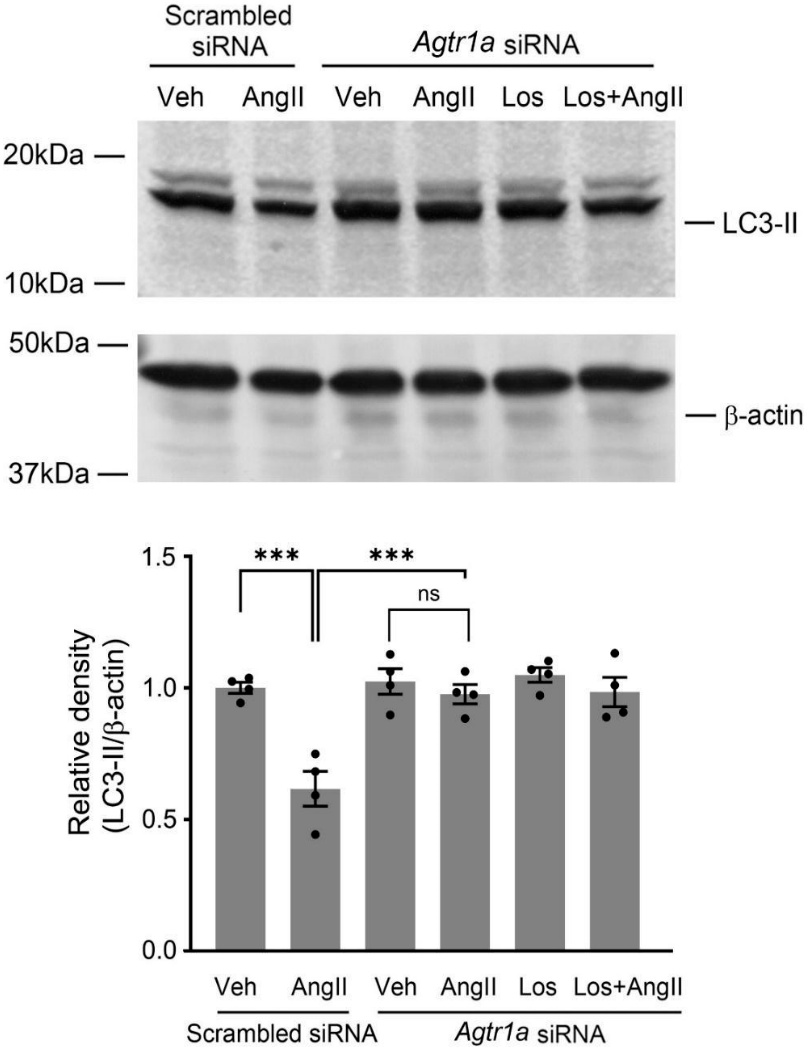

Supplementary Figure S7. LC3-II protein expression in VSMCs transfected with either scrambled or *Agtr1a* siRNA in VSMCs. VSMCs were transfected with siRNAs (48 hours) and then treated with vehicle (Veh) or Ang II (Ang, 100 nmol/L) for 6 hours, prior to the end of 48 hour-transfection in the absence or presence of the AT_1R antagonist, losartan (Los) (10 μmol/L), added 30 minutes prior to the addition of Ang II. LC3-II protein expressions were quantified and expressed as the ratio of LC3-II over β-actin densities, then normalized by the controls (0 hour or 0 mol/L). n = 4/Group, *** $p < 0.001$, ns, not significant. One-way ANOVA, Bonferroni multiple comparison test.

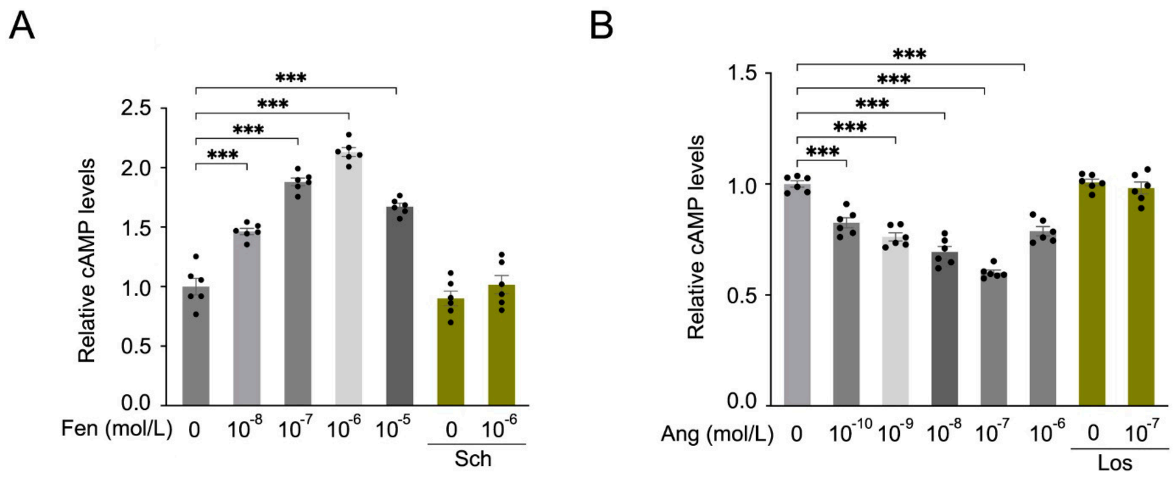

Supplementary Figure S8. Concentration responses of fenoldopam and angiotensin II on cAMP production in VSMCs. (**A**) VSMCs were treated with fenoldopam (Fen) by the indicated concentrations for 15 minutes. Sch, Sch23390 (1 µmol/L). (**B**) VSMCs were incubated with forskolin for 10 minutes before treatment with Ang II by the indicated concentrations for 15 minutes. Ang, Ang II; Los, losartan (10 µmol/L); Veh, vehicle. $n = 6$/Group, *** $p < 0.001$. One-way ANOVA, Bonferroni multiple comparison test.

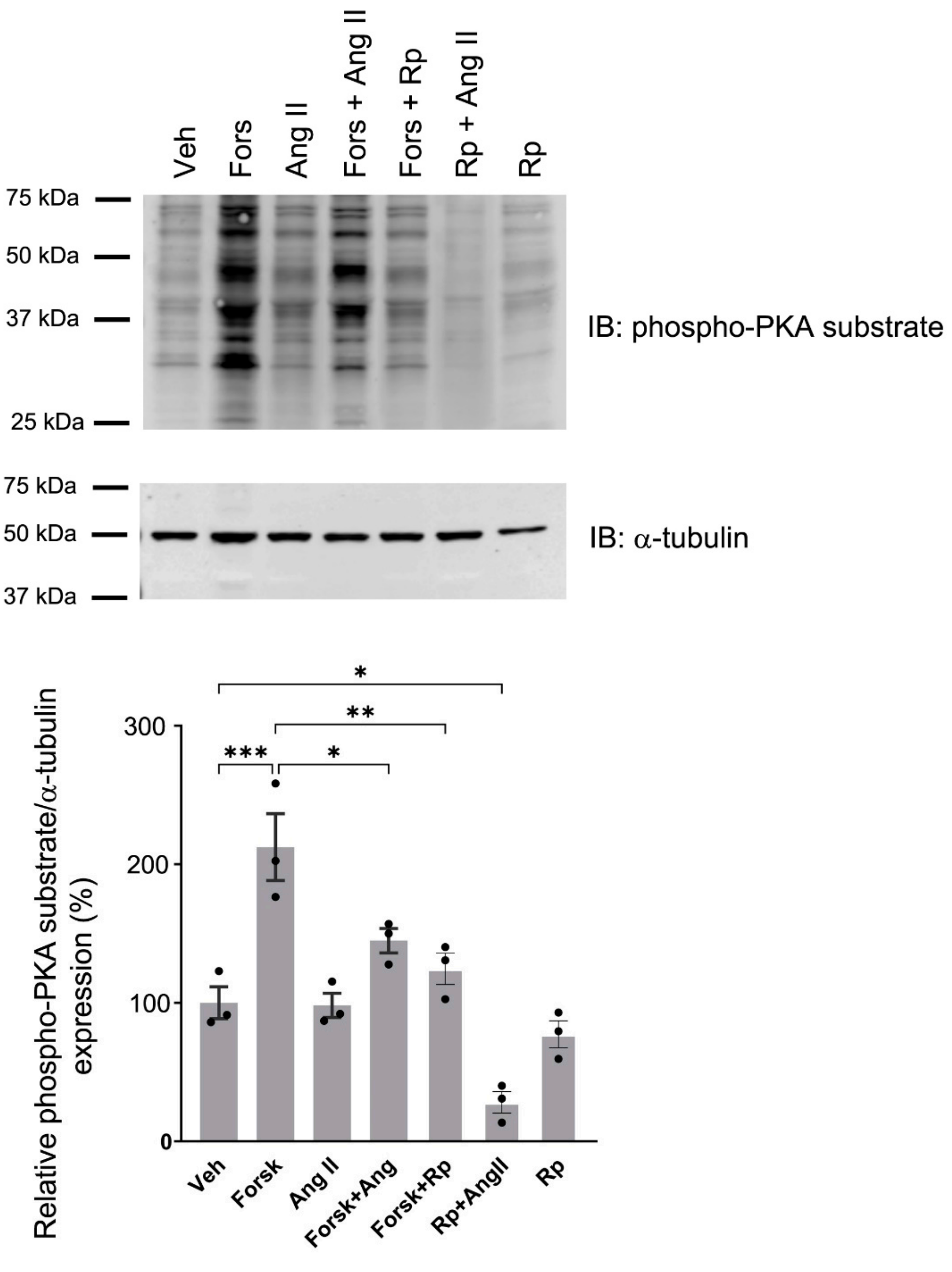

Supplementary Figure S9. Protein expression of phospho-PKA substrate in VSMCs treated with Ang II, Rp-cAMPS, and their combinations. VSMC were exposed to Vehicle, forskolin (10 μM), Ang II (100 nM), Rp-cAMPS (50 μM) and their combinations as indicated for 15 min. For the combination groups, Rp-cAMPS was added 30 min earlier before Ang II or forskolin was

added and then incubated together for 15 min. Ang II, angiotensin II; Fors, forskolin; Rp, Rp-cAMPS. The α-tubulin blot served as the sample loading control. The total band intensities of phospho-PKA substrates between 25 kDa and 75 kDa were quantified over their corresponding α-tubulin band intensities. The average band intensity ratio of phospho-PKA substrate/α-tubulin of the vehicle group was set at 100%. $n = 3$/Group, * $p < 0.05$, ** $p < 0.01$, *** $p < 0.001$. One-way ANOVA, Bonferroni multiple comparison test.

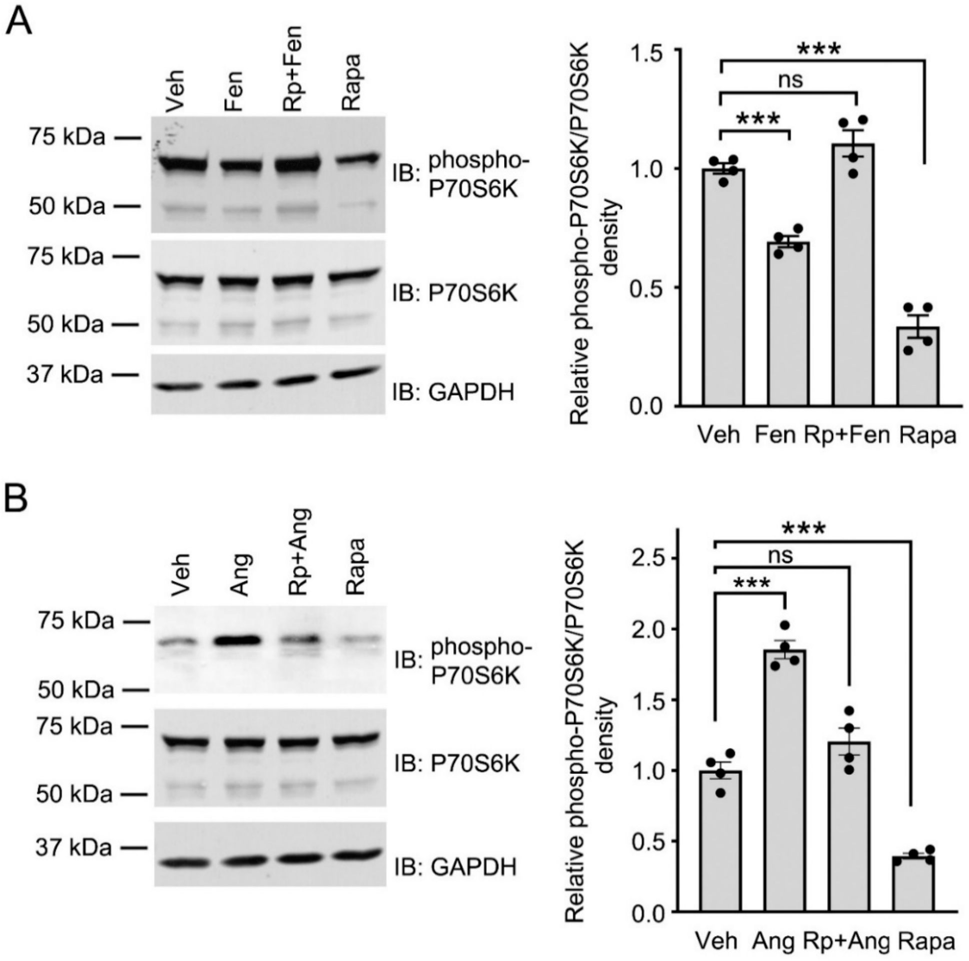

Supplementary Figure S10. Fen- and Ang II-mediated mTOR regulation is cAMP dependent in VSMCs. (**A**) Fen (1 μmol/L, 6 hours) decreased the phosphorylation of P70S6K (a direct substrate of mTOR), which was inhibited by Rp-cAMPS (Rp, 50 μmol/L, 6 hours). Rapamycin (Rapa, 100 nmol/L, 6 hours), a positive control, decreased the phosphorylation of P70S6K. $n = 4$/Group, *** $P<0.001$, ns, not significant. One-way ANOVA, Bonferroni multiple comparison test. (**B**) Ang II (100 nmol/L, 6 hours) increased the phosphorylation of P70S6K, which was inhibited by Rp-cAMPS (Rp, 50 μmol/L, 6 hours). Rapamycin (Rapa, 100 nmol/L, 6 hours), a positive control, decreased the phosphorylation of P70S6K. $n = 4$/Group, *** $p < 0.001$, ns, not significant. One-way ANOVA, Bonferroni multiple comparison test.

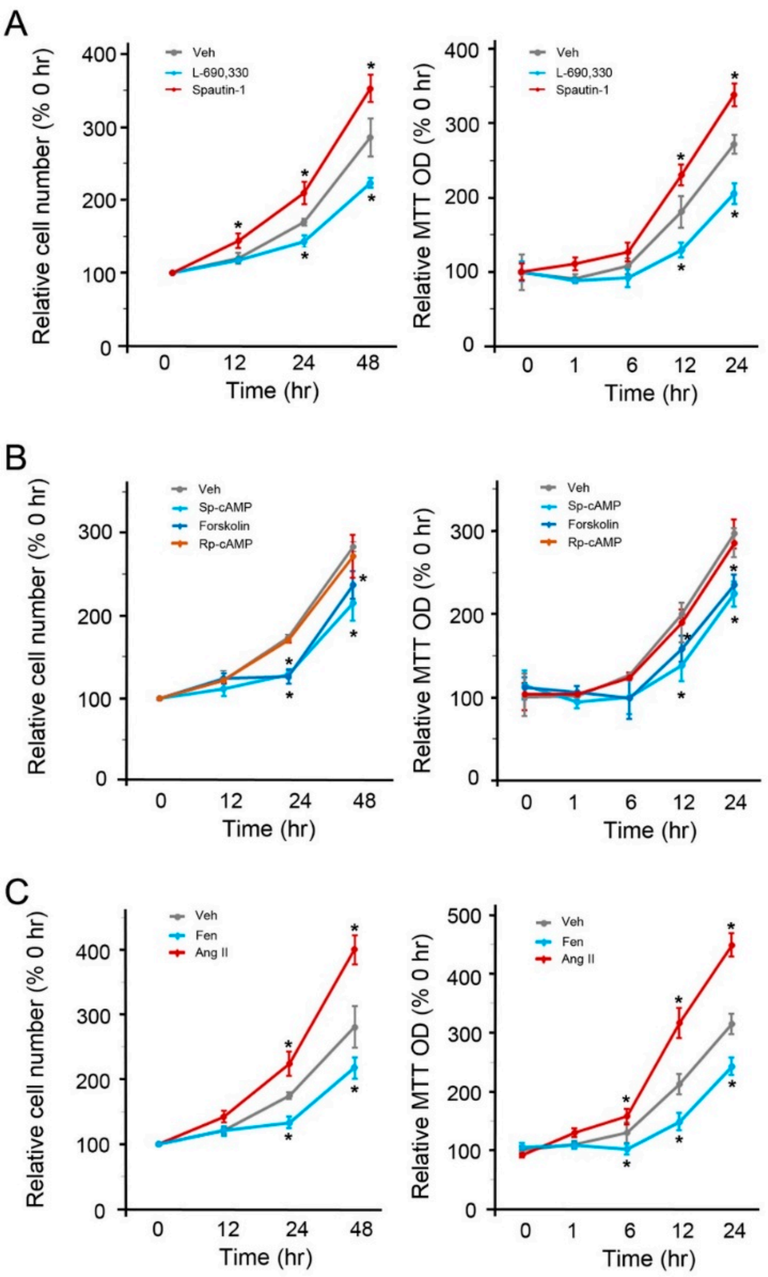

Supplementary Figure S11. Analysis of VSMC proliferation by cell counting (left) and MTT assay (right). (**A**) VSMCs were treated with vehicle (Veh), L-690,330, an autophagy inducer (10 µmol/L), or spautin-1, an autophagy inhibitor (10 µmol/L), at the indicated times. (**B**) VSMCs were treated with vehicle (Veh), Sp-cAMPS, a PKA activator (1.0 µmol/L), forskolin (10 µmol/L), an adenylyl cyclase activator, or Rp-cAMPS (50 µmol/L), a PKA

inhibitor at the indicated times. (**C**) VSMCs were treated with vehicle (Veh), Fen (1 μmol/L), or Ang II (100 nmol/L) at the indicated times. Values (100 % at 0 time) are shown, using Veh-treated cells at their initial time point (0 hour) for normalization. (**A** – **C**) Data are mean ± standard deviation, $n = 3$/Group, * $p < 0.05$ vs Veh. Two-way ANOVA, Bonferroni multiple comparison test.

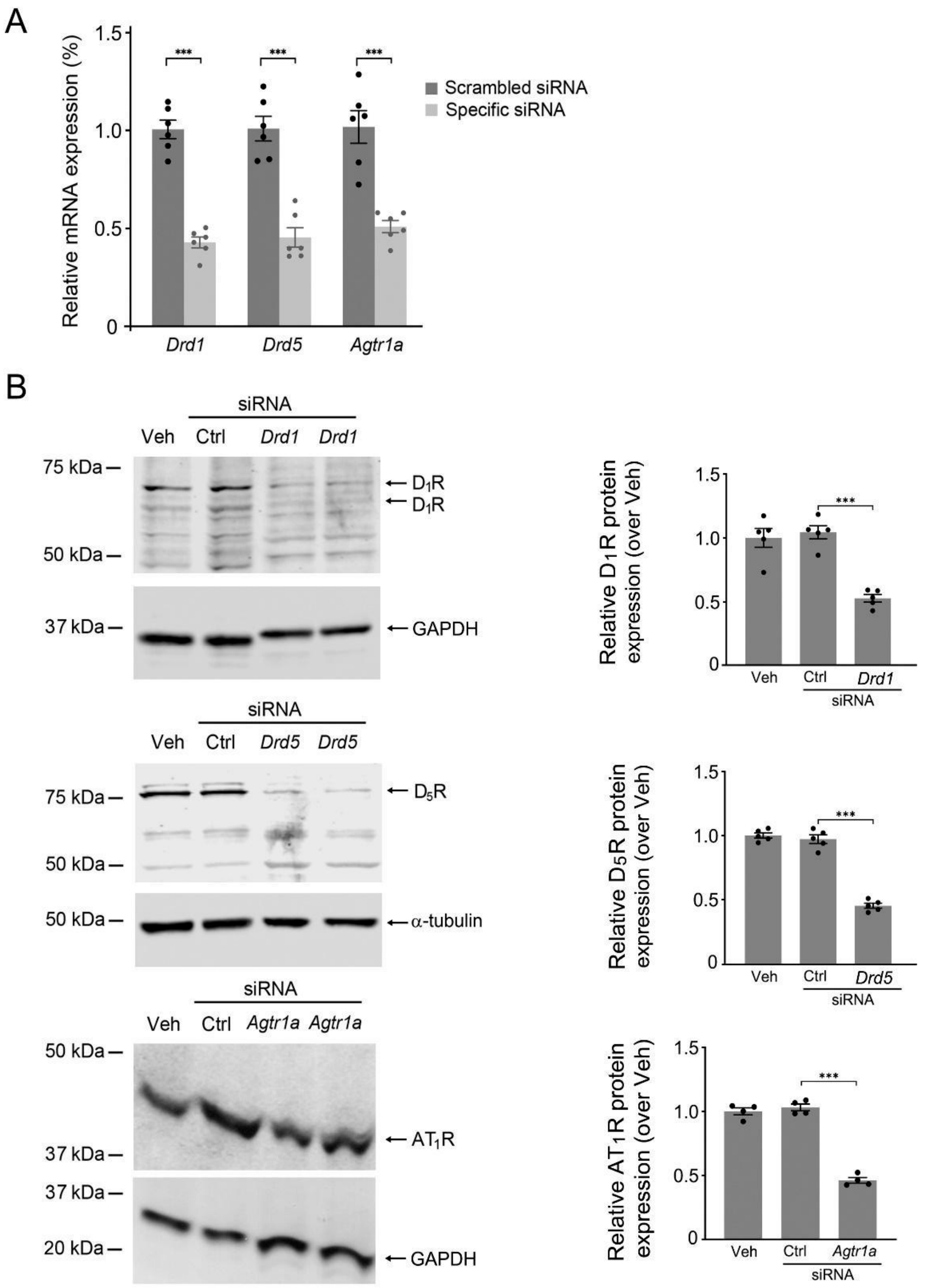

Supplementary Figure S12. Effects of *Drd1*, *Drd5*, and *Agtr1a* siRNAs transfected into VSMCs. (**A**) mRNA expression was quantified by qRT-PCR. VSMCs were transfected with *Drd1*, *Drd5*, and *Agtr1a* siRNAs or scrambled siRNA. $n = 6$/Group, *** $p < 0.001$ vs scrambled

siRNA. One-way ANOVA, Bonferroni multiple comparison test. (**B**) Protein expression was quantified by immunoblotting. VSMCs were transfected with *Drd1*, *Drd5*, *Agtr1a* siRNAs, or scrambled siRNA (Ctrl, control), as in Figure S1A. The cell lysates were electrophoresed (SDS-PAGE) and the proteins, transferred onto nitrocellulose membranes, were immunoblotted with specific anti-D_1R, anti-D_5R, and anti-AT_1R antibodies. Veh, vehicle (RNase-free water); Ctrl, control (scrambled siRNA); IB, immunoblot. One set of blots from four independent experiments are shown. The densities of D_1R, D_5R, or AT_1R immunoblots from Veh, Ctrl (scrambled siRNA), and average of duplicated specific siRNA immunoblots were quantified and expressed as the ratio of D_1R, D_5R, or AT_1R over their individual loading controls and then normalized with Veh group. n = 4-5/Group, *** $p < 0.001$. One-way ANOVA, Bonferroni multiple comparison test.

Supplementary Table S1. Primary antibodies used in this study

Antibody	Source	Cat. No.	Working condition	Note
LC3-II	Cell Signaling Technology	3868	1:500	Monoclonal Rabbit
Beclin-1	Cell Signaling Technology	3495	1:500	Monoclonal Rabbit
P70S6K	Cell Signaling Technology	9202	1:500	Polyclonal Rabbit
Phospho-P70S6K	Cell Signaling Technology	9205	1:500	Polyclonal Rabbit
Phospho-PKA substrate (RRXS*/T*)	Cell Signaling Technology	9624	1:500	Monoclonal Rabbit
D_1R	In-house	NA	1:200	Polyclonal Rabbit
D_5R	In-house	NA	1:250	Chicken IgY
AT_1R	GeneTex	GTX89149	1:200	Polyclonal goat
α-tubulin	Abcam	ab4074	1:2000	Polyclonal Rabbit
GAPDH	Abcam	ab8245	1:2000	Monoclonal mouse
α-actin	Millipore Sigma	CBL171	1:500	Monoclonal mouse
β-actin	Millipore Sigma	A1978	1:5000	Monoclonal mouse
